# Prebiotic administration modulates gut microbiota and faecal short-chain fatty acid concentrations but does not prevent chronic intermittent hypoxia-induced apnoea and hypertension in adult rats

**DOI:** 10.1016/j.ebiom.2020.102968

**Published:** 2020-08-30

**Authors:** Karen M. O'Connor, Eric F. Lucking, Thomaz F.S. Bastiaanssen, Veronica L. Peterson, Fiona Crispie, Paul D. Cotter, Gerard Clarke, John F. Cryan, Ken D. O'Halloran

**Affiliations:** a Department of Physiology, School of Medicine, College of Medicine & Health, University College Cork, Cork, Ireland; b Department of Anatomy & Neuroscience, School of Medicine, College of Medicine & Health, University College Cork, Cork, Ireland; c APC Microbiome Ireland, University College Cork, Cork, Ireland; d Teagasc Food Research Centre, Moorepark, Fermoy, County Cork, Ireland; e Department of Psychiatry and Neurobehavioural Science, School of Medicine, College of Medicine & Health, University College Cork, Cork, Ireland

**Keywords:** Chronic intermittent hypoxia, Prebiotics, Apnoea, Hypertension, Autonomic dysfunction, Neurochemistry, Short-chain fatty acids, Vagus, Microbiota, AUC, area under the curve, BH, Benjamini-Hochberg, CIH, chronic intermittent hypoxia, DA, dopamine, DOPAC, 3,4-Dihydroxyphenylacetic acid, Dia, diaphragm, EMG, electromyogram, ETCO_2_, end-tidal carbon dioxide, FDR, false discovery rate, *f*_r_, respiratory frequency, FiCO_2_, fractional inspired carbon dioxide concentration, F_i_O_2_, fractional inspired oxygen concentration, GABA, gamma-Aminobutyric acid, GBM, gut-brain modules, GMM, gut-metabolic modules, HFD, high-fat diet, HSD, high-salt diet, HVA, homovanillic acid, IFN, interferon, IL, interleukin, KC/GRO, keratinocytechemoattractant/growth-related oncogene, KEGG, Kyoto Encyclopedia of Genes and Genomes, _L_-DOPA, _L_-3,4-dihydroxyphenylalanine, LSD, least significant difference, NA, noradrenaline, NaCN, sodium cyanide, NTS, nucleus tractus solitarius, OSA, Obstructive sleep apnoea, PBG, phenylbiguanide, PCA, principal component analysis, PCoA, Principal coordinates analysis, PaCO_2_, partial pressure of arterial carbon dioxide, PaO_2_, partial pressure of arterial oxygen, PREB, prebiotic, SaO_2_, arterial oxygen saturation, SDB, sleep-disordered breathing, SD1, short-term respiratory timing variability, SD2, long-term respiratory timing variability, T_e_, expiratory time, T_i_, inspiratory time, TMAO, trimethylamine N-oxide, TNF-α, tumor necrosis factor-α, T_tot_, total breath duration, *V*co_2_, carbon dioxide production, *V*I, minute ventilation, *V*_I_/*V*co_2_, ventilatory equivalent for CO_2_, *V*o_2_, oxygen consumption, V_T_, tidal volume, V_T_/T_i_, mean inspiratory flow, 5-HIAA, 5-hydroindoleacetic acid, 5-HT, 5-hydroxytryptamine (serotonin), 5-HT_3_, 5-hydroxytryptamine type 3

## Abstract

**Background:**

Evidence is accruing to suggest that microbiota-gut-brain signalling plays a regulatory role in cardiorespiratory physiology. Chronic intermittent hypoxia (CIH), modelling human sleep apnoea, affects gut microbiota composition and elicits cardiorespiratory morbidity. We investigated if treatment with prebiotics ameliorates cardiorespiratory dysfunction in CIH-exposed rats.

**Methods:**

Adult male rats were exposed to CIH (96 cycles/day, 6.0% O_2_ at nadir) for 14 consecutive days with and without prebiotic supplementation (fructo- and galacto-oligosaccharides) beginning two weeks prior to gas exposures.

**Findings:**

CIH increased apnoea index and caused hypertension. CIH exposure had modest effects on the gut microbiota, decreasing the relative abundance of *Lactobacilli* species, but had no effect on microbial functional characteristics. Faecal short-chain fatty acid (SCFA) concentrations, plasma and brainstem pro-inflammatory cytokine concentrations and brainstem neurochemistry were unaffected by exposure to CIH. Prebiotic administration modulated gut microbiota composition and diversity, altering gut-metabolic (GMMs) and gut-brain (GBMs) modules and increased faecal acetic and propionic acid concentrations, but did not prevent adverse CIH-induced cardiorespiratory phenotypes.

**Interpretation:**

CIH-induced cardiorespiratory dysfunction is not dependant upon changes in microbial functional characteristics and decreased faecal SCFA concentrations. Prebiotic-related modulation of microbial function and resultant increases in faecal SCFAs were not sufficient to prevent CIH-induced apnoea and hypertension in our model. Our results do not exclude the potential for microbiota-gut-brain axis involvement in OSA-related cardiorespiratory morbidity, but they demonstrate that in a relatively mild model of CIH, sufficient to evoke classic cardiorespiratory dysfunction, such changes are not obligatory for the development of morbidity, but may become relevant in the elaboration and maintenance of cardiorespiratory morbidity with progressive disease.

**Funding:**

Department of Physiology and APC Microbiome Ireland, University College Cork, Ireland. APC Microbiome Ireland is funded by Science Foundation Ireland, through the Government's National Development Plan.

**Research in context**

**Evidence before this study**

Studies to date highlight a contributory role of perturbations to microbiota-gut-brain axis signalling in the manifestation of obstructive sleep apnoea (OSA)-induced cardiorespiratory dysfunction. There is a growing interest in developing strategies to manipulate the microbiota as a potential therapeutic intervention in the treatment of cardiorespiratory disease.

**Added value of this study**

Chronic intermittent hypoxia (CIH) exposure in rats, which caused increased apnoea index and hypertension, had modest effects on the gut microbiota. Faecal short-chain fatty acid (SCFA) concentrations, pro-inflammatory cytokine concentrations and brainstem neurochemistry were unaffected by exposure to CIH. Prebiotic administration modulated gut microbiota composition, diversity and function, but did not prevent adverse CIH-induced cardiorespiratory phenotypes.

**Implications of all the available evidence**

We revealed for the first time using whole-metagenome shotgun sequencing that *Lactobacilli* species are decreased in CIH-exposed rats, but gut microbial functional characteristics are unaltered. Faecal SCFA concentrations were not altered by CIH exposure. Prebiotics modulated gut-metabolic and gut-brain modules and increased faecal SCFAs, but these changes were not sufficient to prevent CIH-induced cardiorespiratory dysfunction in our model. Our results contribute to a growing interest in the role of the microbiota-gut-brain axis in OSA-related morbidities.

Alt-text: Unlabelled box

## 1 Introduction

Obstructive sleep apnoea (OSA), the most common form of sleep-disordered breathing (SDB), is recognised as a major worldwide health crisis with devastating consequences for integrative body systems [1]. OSA is characterised by repetitive collapse of the pharyngeal airway during sleep, with episodic oxygen fluctuations culminating in recurrent exposure to chronic intermittent hypoxia (CIH). It is now apparent that exposure to CIH has adverse effects on the cardiorespiratory control network and is recognised as a major driver of OSA-related morbidities [2], [3], [4], [5], [6], [7], [8].

Studies have recurrently implicated the carotid bodies, the dominant peripheral oxygen sensors, in the manifestation of CIH-induced cardiorespiratory dysfunction [5, 6, [9], [10], [11], [12]]. However, exposure to CIH elicits cardiorespiratory and autonomic disturbances in guinea-pigs with hypoxia-insensitive carotid bodies [13, 14], revealing that sites beyond the carotid bodies can contribute to the manifestation of CIH-induced cardiorespiratory and autonomic disturbances. It is known that CIH-induced plasticity also occurs at other key sites of the cardiorespiratory control circuit, including the nucleus tractus solitarius (NTS), pre-Bötzinger complex, ponto-medullary network and paraventricular nucleus of the hypothalamus [15], [16], [17], [18], [19], [20]. More recently, studies have described effects of CIH on other peripheral sites including the gut microbiota [12, 14, [21], [22], [23], [24], [25], [26]].

The microbiota-gut-brain axis plays a critical regulatory role in physiological systems [27]. Dysregulated microbiota-gut-brain axis signalling affects homoeostatic neurocontrol networks manifesting in pathophysiological behaviours and brain functions [28], [29], [30]. Recent studies extend this concept to cardiorespiratory control [14, 31]. There is considerable interest in the modulatory role of the gut microbiota and gut microbiota metabolites, particularly short-chain fatty acids (SCFAs), in cardiovascular and autonomic function [32], [33], [34], [35]. Proliferation of lactate-producing, as well as diminished butyrate- and acetate-producing taxa is evident in hypertensive models [32, 34, 36, 37]. Hypertensive donor faeces transferred to normotensive animals leads to the development of hypertension in recipient animals [34, 37, 38]. Moreover, in a rat model of SDB, prebiotic administration stimulates the expansion of beneficial commensal microbiota augmenting several SCFA-producing taxa, restoring caecal acetate concentrations and preventing the establishment of hypertension [33]. Chronic acetate administration into the caecum of OSA + high-fat diet (OSA+ HFD) rats prevents the development of high blood pressure [33]. Additionally, butyrate treatment in angiotensin-II-induced hypertensive mice as well as spontaneously hypertensive rats prevented the establishment of hypertension [32, 39].

In rat models, disruption of the gut microbiota using antibiotic administration, faecal microbiota transfer or pre-natal stress results in altered ventilatory responses to hypoxic and hypercapnic chemostimulation [29, 31]. Respiratory frequency response to hypercapnic chemostimulation correlated with altered bacterial genera in adult rats with antecedent pre-natal stress [29]. Several genera, predominantly from the Firmicutes phylum correlated with brainstem neuromodulators crucial in the control of breathing [31]. Exposure to CIH dysregulates cardiorespiratory control in guinea-pigs resulting in aberrant phenotypes including altered autonomic control of heart rate, decreased respiratory variability and prevalence of protective sighs and brainstem noradrenaline concentrations, as well as disturbed gut microbiota indicating that aberrant gut microbiota may at least partly contribute to cardiorespiratory and autonomic malaise in CIH-exposed guinea-pigs [14].

Collectively, these studies and others [25, 40] highlight a contributory role of perturbations to microbiota-gut-brain axis signalling in the manifestation of OSA-induced cardiorespiratory dysfunction. There is a growing interest in developing strategies to manipulate the microbiota as a potential therapeutic intervention in the treatment of cardiorespiratory disease. Rodent and human studies have revealed that prebiotic administration has positive impacts on brain neurochemistry and functions [30, [41], [42], [43], [44]]. Moreover, prebiotic feeding prevented the development of hypertension in a rat model of OSA [33]. Therefore, we assessed cardiorespiratory physiology and gut microbiota composition and diversity in adult rats following exposure to normoxia (Sham) or CIH. We hypothesised that there would be evidence of cardiorespiratory and autonomic dysfunction and altered gut microbiota in CIH-exposed rats. We examined the effects of prebiotic fibre supplementation to test the hypothesis that manipulation of the gut microbiota ameliorates or prevents the deleterious effects of exposure to CIH on cardiorespiratory physiology. We performed whole-genome shotgun sequencing in an attempt to identify microbial patterns that underscore cardiorespiratory homoeostasis and dysfunction.

## 2 Materials and methods

### 2.1 Ethical approval

Procedures on live animals were performed in accordance with European directive 2010/63/EU under authorisation from the Government of Ireland Department of Health (B100/4498) and Health Products Regulatory Authority (AE19130/P070). Ethical approval was obtained from University College Cork (AEEC #2013/035; #2017/023) and procedures were carried out in accordance with guidelines laid down by University College Cork's Animal Welfare Body.

### 2.2 Experimental animals

Eight- to ten-week old adult male Sprague Dawley rats (*n* = 72; purchased from Envigo, UK) were housed as age-matched pairs in standard rat cages. Rodents had *ad libitum* access to standard rat chow and were housed under a 12-hr light: 12-hr dark cycle.

### 2.3 Prebiotic administration

Eight-week old rats (*n* = 24) were randomly allocated to receive equal concentrations of prebiotic fibres in the drinking water (PREB; 7.5 g/L of galactooligossaccharides and fructooligosaccharides; source: Healy group, Tallaght, Dublin, Ireland) with *ad libitum* access for 4-weeks to promote the growth of beneficial host microbiota. Concentrations and prebiotics (galactooligossaccharides and fructooligosaccharides) were chosen based on results from previous studies [45], [46], [47]. After 2 weeks of PREB treatment, a subset of rats were exposed to CIH (see Section 2.4) for the final 2 weeks creating two groups: Sham+PREB (*n* = 12) and CIH+PREB (*n* = 12).

### 2.4 Chronic intermittent hypoxia rat model

Ten-week old rats (*n* = 48) were randomly assigned to one of two groups, each receiving water (vehicle (VEH)): Sham+VEH (*n* = 24) and CIH+VEH (*n* = 24). CIH exposed rats were placed in chambers wherein ambient oxygen concentration was tightly regulated using a dynamic oxygen/nitrogen controller (Oxycycler™; Biospherix, New York, NY, USA). CIH exposure was comprised of 96 cycles of 90 secs of exposure to hypoxia (nadir, FiO_2_ = 0.06, balance N_2_) and 180 secs of exposure to normoxia (FiO_2_ =0.21; balance N_2_), over 8 h during the light phase for 14 consecutive days. Animals were studied on the day subsequent to the last day of CIH exposure. Concurrently, rats assigned to the Sham group were exposed to room air (normoxia) in the same room with similar environmental cues for the duration of the study.

### 2.5 Assessment of respiratory flow in rats during quiet rest

#### 2.5.1 Whole-body plethysmography

During quiet rest, whole-body plethysmography (DSI, St. Paul, Minnesota, USA) was used to record respiratory flow signals during quiet rest. Animals were placed into custom plethysmograph chambers (601–1427–001 PN, DSI) with a room air flow rate maintained at 3l/min. Animals were allowed to acclimate for 30–90 min to encourage habituation to the new surroundings. Paired contemporaneous observations were performed during light hours in Sham+VEH (*n* = 12) *versus* CIH+VEH (*n* = 12) and subsequently Sham+PREB (*n* = 12) *versus* CIH+PREB (*n* = 12) using a pair of plethysmograph chambers.

#### 2.5.2 Metabolic measurements

O_2_ consumption (VO_2_) and CO_2_ production (VCO_2_) were measured in rodents throughout the experimental protocol (O_2_ and CO_2_ analyser; AD Instruments, Colorado Springs, CO, USA) as previously described [14, 31, 48, 49].

#### 2.5.3 Experimental protocol

Once the acclimation period was complete and animals were confirmed to be at quiet rest, baseline parameters were recorded during a 10–15 min steady-state normoxia period (FiO_2_ = 0.21; balance N_2_). This was followed by a 10 min poikilocapnic hypoxia challenge (FiO_2_=0.10; balance N_2_). Normoxia was subsequently restored in each chamber to re-establish stable baseline breathing. Thereafter, a second baseline period was recorded followed by a 10 min hypercapnia challenge (FiCO_2_ = 0.05; balance O_2_). Subsequently, a third normoxic baseline period was recorded. Rats were then exposed to a 10 min hypoxic hypercapnic challenge (FiO_2_ = 0.10; FiCO_2_ =0.05, balance N_2_).

#### 2.5.4 Data analysis for whole-body plethysmography

Respiratory parameters including tidal volume (*V*_T_), respiratory frequency (*f*_R_), minute ventilation (*V*_I_), expiratory time (T_e_) and inspiratory time (T_i_) were recorded on a breath-by-breath basis for analysis (FinePointe software Buxco Research Systems, Wilmington, NC, USA). Artefacts relating to animal movement and sniffing in respiratory flow recordings were omitted from analysis. A single baseline period during normoxia was determined by averaging the three baseline recording epochs to determine resting steady-state respiratory and metabolic parameters. Ventilatory and metabolic data were averaged and reported for the final 5 min of acute poikilocapnic hypoxia, hypercapnia and hypoxic hypercapnia allowing sufficient time for gas mixing in the custom plethysmograph chambers. Data are expressed as absolute change from baseline values. Respiratory flow recordings were assessed for the occurrence of augmented breaths (sighs) during normoxia, poikilocapnic hypoxia and hypercapnia, as well as the frequency of apnoea events (post-sigh and spontaneous apnoeas) during normoxia as previously described [50]. The criterion for an apnoea was a pause in breathing greater than two consecutive missed breaths. Apnoea data are expressed as apnoea index (apnoea events per hour), combining post-sigh and spontaneous apnoeas. A sigh was defined as an augmented breath, double the amplitude of the average *V*_T_. The frequency and amplitude of sighs were determined. Poincaré plots expressing breath-to-breath (BB*n*) *versus* subsequent breath-to-breath interval (BB*n+1*) were extrapolated allowing for determination of short- (SD1) and long-term (SD2) respiratory timing variability during steady-state baseline breathing. V_T_, *V*_I_, *V*_T_/T_i_, *V*O_2_ and *V*CO_2_ were normalised per 100 g body mass.

### 2.6 Assessment of cardiorespiratory parameters under urethane anaesthesia

#### 2.6.1 Surgical protocol and cardiorespiratory measures

Following whole-body plethysmography, cardiorespiratory parameters were assessed in Sham+VEH, CIH+VEH, Sham+PREB and CIH+PREB rats (*n* = 11–12 per group) under urethane anaesthesia (1.5 g/kg i.p.; 20% w/v) following isoflurane induction (5% by inhalation in room air). Throughout the surgical and experimental protocol, the depth of anaesthesia was carefully assessed by monitoring reflex responses to tail/paw pinch and the corneal reflex. Supplemental doses of anaesthetic were given as required. Rodents were placed in a supine position on a homoeothermic blanket system (Harvard Apparatus, Holliston, MA, USA) and a rectal temperature probe and heating pad used to maintain core temperature at 37 °C. A mid-cervical tracheotomy was performed, followed by intravenous (i.v.) cannulation of the right jugular vein for infusion of supplemental anaesthetic and drugs. The carotid (*n* = 22)/femoral artery (*n* = 1) was cannulated for the recording of arterial blood pressure and the withdrawal of blood samples for arterial blood gas, pH and electrolyte analysis (i-STAT; Abbott Laboratories Ltd). Using a foot clip, peripheral oxygen saturation (SpO_2_; Starr Life Sciences, PA, USA) was determined and maintained above 95% via a bias flow of supplemental O_2_ passing the tracheal cannula sourced from a gas mixing system (GSM-3 Gas Mixer; CWE Inc.). A pneumotachometer (Hans Rudolf) and a CO_2_ analyser (microCapStar End-Tidal CO_2_ analyser; CWE Inc., USA) were connected to the tracheal cannula to measure tracheal flow and end-tidal CO_2_ (ETCO_2_), respectively. Diaphragm electromyogram (EMG) activity was continuously measured using a concentric needle electrode (26 G; Natus Manufacturing Ltd., Ireland). Signals were amplified (x5,000), filtered (band pass; 500–5000 Hz) and integrated (50 ms time constant; Neurolog system, Digitimer Ltd., UK). Data were digitised via a PowerLab-LabChart v7 (ADInstruments) data acquisition system.

#### 2.6.2 Experimental protocol

An arterial blood sample was obtained from each animal following a 30 min stabilisation period, after which, a minimum 10 min baseline recording period was observed for assessment of baseline parameters (FiO_2_ = 0.25–0.40; balance N_2_). The rats were exposed to a poikilocapnic hypoxia challenge (FiO_2_ = 0.12, balance N_2_) for 5 min, followed by a recovery period. Animals were then exposed to a 5 min hypoxic hypercapnic challenge (FiO_2_ = 0.12, FiCO_2_ = 0.05, balance N_2_). Following a recovery period, sodium cyanide (NaCN; 200 μg/kg i.v.) was administered to evoke carotid body dependant increases in ventilation. After an adequate recovery period and removal of the pneumotachometer, a second arterial blood sample was taken. Next, the serotonin type 3 (5-HT_3_) receptor agonist phenylbiguanide (PBG; 25 μg/kg i.v.) was administered to stimulate pulmonary vagal afferent C-fibres [51, 52] eliciting the classic pulmonary chemoreflex. Successively, phenylephrine (50 μg/kg i.v.), sodium nitroprusside (50 μg/kg i.v.), atenolol (2 mg/kg i.v.), propranolol (1 mg/kg i.v.) and hexamethonium (25 mg/kg i.v.) were administered to assess cardiovascular responses to pharmacological manipulation with sufficient recovery periods allowed between each pharmacological challenge. Animals were euthanised by urethane (i.v) overdose. One animal (Sham+PREB, *n* = 1) presented with uncharacteristically poor ventilatory and cardiovascular responses throughout the experimental protocol; this animal was excluded from data analysis. In all animals, blood was collected, prepared in 3% Na_2_EDTA (disodium salt dehydrate) and centrifuged. Plasma was snap frozen in liquid nitrogen for subsequent analysis of corticosterone and pro-inflammatory cytokine concentrations. Whole brains were removed, separated into pons and medulla oblongata, frozen in isopentane at −80 °C and stored at −80 °C until subsequent analysis by high-performance liquid chromatography. The lungs were removed and weighed and were allowed to air dry at 37 °C for at least 48 hrs before being re-weighed to provide an index of pulmonary oedema. The caecum was removed, weighed and caecal contents were removed and snap frozen in liquid nitrogen for whole-genome shotgun sequencing. Faeces were removed from the colon for the assessment of SCFA concentrations by gas chromatography. The heart was removed, and the right ventricle was separated from the left ventricle + septum and each were weighed separately.

#### 2.6.3 Data analysis of cardiorespiratory parameters in anaesthetised rats

Baseline parameters were averaged over 10 min of stable recording and data are presented as absolute values. For cardiorespiratory and EMG responses to poikilocapnic hypoxia and hypoxic hypercapnia the average of the last minute of recordings was determined and data were compared with the 1 min pre-challenge baseline. Data for drug challenges were averaged into 3 or 5 second bins and the peak cardiorespiratory responses to NaCN, PBG, phenylephrine, sodium nitroprusside, atenolol, propranolol and hexamethonium administration were determined and compared to the respective 1 min pre-challenge baseline. Maximum apnoea and post-apnoea tachypnoea in response to PBG are expressed as the duration of the apnoea or tachypnoea period normalised in each trial to the average cycle duration determined during the 30 s pre-challenge baseline period. All cardiorespiratory responses to chemostimulation and drug administration are expressed as percent change from the preceding baseline values.

### 2.7 Brainstem monoamine concentrations

#### 2.7.1 High-performance liquid chromatography (HPLC) coupled to electrochemical detection for the measurement of brainstem monoamine concentrations

Pons (*n* = 11–12/group) and medulla oblongata (*n* = 11–12/group) tissues were sonicated (Bandelin Sonopuls HD 2070) in 1 ml of chilled mobile phase, spiked with 2 ng/20μl of a N-methyl 5-HT as internal standard. Brainstem monoamine, precursor and metabolite concentrations were measured as previously described [14, 31]. Noradrenaline (NA), dopamine (DA), serotonin (5-HT), and monoamine metabolites and precursor, 5-hydroxyindoleacetic acid (5-HIAA), homovanillic acid (HVA), 3,4-Dihydroxyphenylacetic acid (DOPAC) and _l_-3,4 dihydroxyphenylalanine (_L_-DOPA) were identified by their characteristic retention times and concentrations determined by comparison against standard injections run at regular intervals during sample analysis.

#### 2.7.2 Data analysis

Class-VP5 software was used to process chromatographs. Concentrations (ng/g) of monoamines, precursors and metabolites in each sample were determined using analyte:internal standard peak response ratios.

### 2.8 Plasma and brainstem pro-inflammatory cytokine concentrations

#### 2.8.1 Brainstem tissue homogenisation and protein quantification

A separate cohort of rats (Sham, *n* = 12; CIH, *n* = 12) were euthanised by pentobarbitone (i.v.) overdose and whole brains were removed. The pons and medulla oblongata were separated from the brain, frozen in isopentane at −80 °C and stored at −80 °C until subsequent determination of brainstem cytokine concentrations. Pons and medulla oblongata tissue (Sham, *n* = 12; CIH, *n* = 12) were weighed and sonicated (1 ml per 100 mg of tissue) in radioimmunoprecipitation assay (RIPA buffer) (10X RIPA, deionised H_2_0, 200Mm sodium fluoride, 100Mm, phenylmethylsulfonylfluoride (PMSF), 1X protease inhibitor cocktail and 1X phosphate inhibitor cocktail). Samples were centrifuged at 10,000 *g* for 15 min at 4 °C, to pellet membranes and nuclei. The protein concentration of each sample was determined using a bicinchoninic acid (BCA) protein quantification assay (Thermo Fisher Scientific) as per the manufacturer's instructions, at a dilution of 1:10.

#### 2.8.2 Multiplex assay for measurement of plasma and brainstem pro-inflammatory cytokines

Concentrations of interleukin (IL)−1β, IL-4, IL-5, IL-10, IL-13, interferon (IFN)-γ, keratinocytechemoattractant/growth-related oncogene (KC/GRO) and tumour necrosis factor (TNF)-α were measured in plasma (all groups; *n* = 11–12/group) as well as pons and medulla oblongata (Sham and CIH only; *n* = 12 each group) supernatants by sandwich immunoassay methods using commercially available detection kits (V-Plex Pro-inflammatory Panel 2 (rat) kit; Meso Scale Discovery, Gaithersburg, USA) as per the manufacturer's instructions. For pons and medulla oblongata tissues, 100 μg of protein sample was loaded per well as previously described [51]. Plates were read using QuickPlex SQ 120 imager and computer (Meso Scale Discovery).

### 2.9 Plasma corticosterone

Plasma samples were thawed and concentrations of corticosterone were determined using commercially available enzyme-linked immunosorbent assay kit according to the manufacturer's instructions (ENZO Life Sciences, UK) using a spectrophotometer (SpectraMax M3, Molecular devices).

### 2.10 Microbiota composition and function

#### 2.10.1 DNA extraction from caecal material

DNA was extracted from caecal material as previously described [53].

#### 2.10.2 Whole-metagenome shotgun sequencing

Whole-metagenome shotgun libraries were prepared in accordance with the Nextera XT DNA Library Preparation Guide from Illumina with the exception that the tagmentation time was increased to 7 min. After indexing and clean-up of the PCR products as described in the protocol, each sample was run on an Agilent bioanalyser high sensitivity chip (Agilent) to determine the size range of the fragments obtained. The concentration of the samples was also determined at this point using a Qubit High Sensitivity Assay (Life-Sciences). Samples were then pooled equimolarly and the final concentration of the pooled library was determined by quantitative PCR using the Kapa Library Quantification kit for Illumina (Roche). The pooled library was then sequenced on the Illumina NextSeq using the 2 × 150 High Output kit according to standard Illumina sequencing protocols.

#### 2.10.3 Metagenomic bioinformatic analysis

Shotgun metagenomic sequence files (BCL, base calls) were converted to fastq format using bcl2fastq version 2.19. Forward and reverse fastq files were processed using KneadData version 0.7.2 from the Huttenhower bioBakery suite [54]. A reference library was created to remove host DNA in Bowtie2 version 2.3.4 from the NCBI rat genome (*Rattus norgegicus,* GCF 000,001,895.5). Quality filtering was performed using the default setting (ex., phred=33) and trimming with Trimmomatic version 0.38–1. Resulting high quality paired-end reads for each sample were then concatenated in KneadData. Kraken2 version 2.0.7-beta was used for taxonomic classification with the standard database. Report files of taxonomic counts for each sample were merged into a single count file using a custom R script and ran in R version 3.5.2.

#### 2.10.4 Functional annotation, gut-brain module and gut-metabolic module analysis

Humann2 was used to generate a table of Kyoto encyclopaedia of Genes and Genomes (KEGG) Orthologues. This table was aggregated into gut-brain modules (GBMs) and gut-metabolic modules (GMMs), pathways of functions performed by the gut microbiome that have the potential to influence the host brain [55, 56], using the omixer-rpmR library for R [57].

### 2.11 Faecal short-chain fatty acid concentrations

SCFA analysis and extraction were carried out by MS-Omics (Vedbaek, Denmark) as follows. Faecal water was prepared by homogenising the faecal samples (approximately 100 mg) in ultrapure water (3 µl/µg). Samples were then vortexed for 2 min followed by centrifugation (5 min, 30,000 *g*, 5 °C). The supernatant was transferred to a centrifuge filter and the filtered samples were used for analysis. The filtrate was acidified using hydrochloride acid, and deuterium labelled internal standards where added. All samples were analysed in a randomized order. Analysis was performed using a high polarity column (Zebron™ ZB-FFAP, GC Cap. Column 30 m x 0.25 mm x 0.25 µm) installed in a gas chromatography (GC; 7890B, Agilent) coupled with a quadropole detector (59977B, Agilent). The system was controlled by ChemStation (Agilent). Raw datasets were converted to netCDF format using Chemstation (Agilent), before the data was imported and processed in Matlab R2014b (Mathworks, Inc.) using the PARADISe software [58]. Data are expressed as absolute concentration in mM.

### 2.12 Statistical analysis

Data (except microbiota data) were assessed for outliers, normal distribution and equality of variances using box-plots, Shapiro-Wilk test and Levene's test, respectfully. Parametric data were statistically compared using independent samples *t*-test and two-way ANOVA followed by Fisher's least significant test for pairwise comparisons, where appropriate. Non-parametric data were statistically compared using Mann-Whitney *U* test and Kruskal-Wallis test followed by Mann-Whitney *U* test for pairwise comparisons, where appropriate. Statistical significance was assumed at *p<*0.05. Bonferroni correction was applied to adjust for multiple comparisons with the exception of microbiota data. Statistical significance for multiple comparisons was accepted at *p<*0.05 divided by the number of comparisons made.

As part of filtering, microbes prevalent in only 5% or fewer samples were excluded. For differential abundance analysis, an additional threshold of 0.5% abundance had to be reached in at least one sample to be considered. When handling compositional data, the compositionally appropriate centred-log ratio (clr) transformation was performed using the ALDEx2 R library in preparation of statistical testing [59]. The ALDEx2 library was also used to test for differentially abundant features, using a pairwise implementation of the *aldex.ttest()* function to compare multiple groups. Benjamini-Hochberg (BH) adjustment procedure was applied with the false discovery rate (FDR) set at 10% to correct for multiple testing in the relative abundance microbiota data The 2D principal component analysis (PCA) plot was constructed using the clr transformed values computed using the ALDEx2 [60] library in R (version 3.6.0) with Rstudio (version 1.1.453), as is appropriate for compositional data [61] using recommended parameters and 1000 permutations. Pairwise PERMANOVA was used to analyse the 2D PCA plot. Alpha diversity indices were statistically analysed using non parametric Mann-Whitney *U* test, followed by Bonferroni-Holm. For correlation analysis between bacterial specie*s* and physiological parameters of interest, Hierarchical All-against-All association testing (HAllA) [62] was used (version 0.8.7) with Spearman correlation as correlation ​metric, medoid as clustering method and *q* < 0.1 as threshold for significance. Microbiota data are expressed as median (IQR). All other data are expressed as mean ± SD or displayed graphically as box and whisker plots (median, IQR and minimum to maximum values). SPSS v25 was used for all other statistical analysis. GraphPad Software v6 (GraphPad Software, San Diego, CA, USA) and R software environment were used to generate graphs. Adobe illustrator CS5 (v15) was used to edit figures.

## 3 Results

### 3.1 Body and tissue weights

CIH exposure and prebiotic administration had a significant effect on body weight (Diet*CIH, F (1, 43) = 5.426, *p =* 0.025, ƞ^2^=0.112, Table 1). The combination of CIH+PREB decreased body weight gain compared with CIH+VEH or Sham+PREB rats; Sham+PREB rats were also lighter than Sham+VEH rats. CIH exposure had no effect on caecum weight but as expected, prebiotic supplementation increased caecum weight. Differences between groups in normalised cardiac ventricle weights relate to changes in body weight (Supplementary Table 1).

**Table 1:** Body and tissue weights.

	Sham+VEH	CIH+VEH	Sham+PREB	CIH+PREB	p-value (Kruskal-Wallis)	p-value (two-way ANOVA)	Sham+VEH v CIH+VEH	CIH+VEH v CIH+PREB	Sham+PREB v CIH+PREB	Sham+VEH v Sham+PREB
Body mass (g)	368 ± 16	346 ± 21	308 ± 27	263 ± 16	N/A	Diet, ***p*<0.0005**; CIH, ***p*<0.0005**; Diet*CIH, ***p*** **=** **0.025**	0.057	**0.0005**	**<0.0005**	**<0.0005**
RV (mg/100 g)	46 ± 7.8	50 ± 6	58 ± 9.1	61 ± 9.1	**<0.0005**	N/A	0.325	**0.004**	0.908	**0.001**
LV (mg/100 g)	235 ± 24	232 ± 19	238 ± 22	236 ± 22	N/A	Diet, *p* = 0.280; CIH, *p* = 0.810; Diet*CIH, *p* = 0.603	–	–	–	–
LV+RV (mg/100 g)	278 ± 18	282 ± 15	299 ± 25	297 ± 29		Diet, ***p*** **=** **0.009**; CIH, *p* = 0.821; Diet*CIH, *p* = 0.569	0.578	0.139	0.806	0.023
Caecum (g/100 g)	0.97 ± 0.27	0.87 ± 0.1	2.1 ± 0.46	2.3 ± 0.7	N/A	Diet, ***p*<0.0005**; CIH, *p* = 0.716; Diet*CIH, *p* = 0.345	0.709	**<0.0005**	0.293	**<0.0005**
Oedema index (% wet weight)	78 ± 1	77 ± 3	79 ± 1	76 ± 5	0.110	N/A	–	–	–	–

BW, body weight; RV, right ventricle; LV, left ventricle; CIH, chronic intermittent hypoxia; PREB, prebiotic; VEH, vehicle. Data are shown as mean ± SD and were statistically compared using two-way ANOVA, followed by Fisher's least significant difference (LSD) *post hoc* where appropriate, or non-parametric Kruskal-Wallis test, followed by Mann-Whitney U test*,* where appropriate. Statistical significance for multiple comparisons was accepted at *p*<0.05 divided by the number of comparisons made, which was four *i.e. p*<0.0125. *p*-values shown in **bold** highlight significant differences.

### 3.2 Baseline ventilation and metabolism in rats during quiet rest

CIH exposure did not affect the majority of respiratory parameters during normoxia. Prebiotic fibre supplementation increased baseline V_I_, V_T_ and V_T_/T_i_ in Sham+PREB and CIH+PREB compared with Sham+VEH and CIH+VEH rats, respectively (Table 2), but the differences related to body weight (Supplementary Table 1). CIH exposure had no effect on VCO_2_ production; CIH+PREB rats had significantly increased VCO_2_ production compared with CIH+VEH rats, but VCO_2_ production in Sham+PREB rats was not different compared with Sham+VEH (Table 2). CIH exposure had no effect on V_I_/VCO_2_ (breathing as a function of metabolism), but prebiotic administration increased V_I_/V*CO*_2_, however *post hoc* analysis revealed no difference between groups (Table 2). In summary, CIH exposure and prebiotic administration had modest effects on ventilation and metabolism during normoxia.

**Table 2:** Baseline ventilation, apnoea, sigh and metabolism in rats during quiet rest.

	Sham+VEH	CIH+VEH	Sham+PREB	CIH+PREB	p-value (Kruskal-Wallis)	p-value (two-way ANOVA)	Sham+VEH v CIH+VEH	CIH+VEH v CIH+PREB	Sham+PREB v CIH+PREB	Sham+VEH v Sham+PREB
*f_R_* (brpm)	82 ± 8	78 ± 9	83 ± 9	81 ± 7	N/A	Diet, *p* = 0.346; CIH, *p* = 0.310; Diet*CIH, *p* = 0.731	–	–	–	–
V_I_ (ml/min/ 100 g)	53 ± 9	54 ± 6	63 ± 9	71 ± 6	**<0.0005**	N/A	0.498	**<0.0005**	0.038	**0.009**
V_T_ (ml/100 g)	0.7 ± 0.1	0.7 ± 0.1	0.8 ± 0.1	0.9 ± 0.1	**<0.0005**	N/A	0.356	**0.001**	0.028	**0.009**
V_T_/T_i_ (ml/s/ 100 g)	2.8 ± 0.6	2.6 ± 0.4	3.1 ± 0.5	3.4 ± 0.3	N/A	Diet, ***p*<0.0005;** CIH, *p* = 0.833; Diet*CIH, *p* = 0.113	0.331	**<0.0005**	0.198	0.093
T_i_ (ms)	254 ± 26	277 ± 21	260 ± 28	268 ± 21	N/A	Diet, *p* = 0.794; CIH, ***p*** **=** **0.032**; Diet*CIH, *p* = 0.307	0.028	0.370	0.402	0.584
T_e_ (ms)	516 ± 74	520 ± 119	515 ± 71	501 ± 54	N/A	Diet, *p* = 0.688; CIH, *p* = 0.825; Diet*CIH, *p* = 0.706	–	–	–	–
VO_2_ (ml/min/ 100 g)	2.7 ± 0.7	2.4 ± 0.8	3.1 ± 1.1	3.3 ± 1.1	N/A	Diet, ***p*** **=** **0.019**; CIH, *p* = 0.815; Diet*CIH, *p* = 0.420	0.467	0.028	0.681	0.251
VCO_2_ (ml/ min/100 g)	1.9 ± 0.3	1.9 ± 0.2	2.0 ± 0.3	2.1 ± 0.3	**0.046**	N/A	0.758	**0.012**	0.525	0.166
V_I_/VCO_2_	28 ± 3	29 ± 4	31 ± 5	34 ± 5	N/A	Diet, ***p*** **=** **0.003**; CIH, *p* = 0.107; Diet*CIH, *p* = 0.603	0.073	0.128	0.015	0.438
Post sigh apnoea (events per hr)	12 ± 6	19 ± 13	9 ± 5	16 ± 9	0.083	N/A	–	–	–	–
Spontaneous apnoea (events per hr)	3.3 ± 5.0	5.5 ± 6.8	3.6 ± 5.2	10.0 ± 7.5	**0.044**	N/A	0.374	0.067	0.022	0.596
Sigh amplitude (ml/100 g)	0.8 ± 0.2	1.2 ± 0.4	1.2 ± 0.2	1.4 ± 0.2	**<0.0005**	N/A	0.023	0.176	0.05	**<0.0005**

*f_R_,* respiratory frequency (brpm, breaths per minute); V_i_, minute ventilation; *V*_T,_ tidal volume; *V*_T_/T_i_, mean inspiratory flow; T_i_, inspiratory time; T_e_, expiratory time; *V*O_2_, oxygen consumption; *V*CO_2,_ carbon dioxide production; V_I_/VCO_2,_ ventilatory equivalent; CIH, chronic intermittent hypoxia; PREB, prebiotic; VEH, vehicle. Data are shown as mean ± SD and were statistically compared using two-way ANOVA, followed by Fisher's least significant difference (LSD) *post hoc* where appropriate, or non-parametric Kruskal-Wallis test, followed by Mann-Whitney U test*,* where appropriate. Statistical significance for multiple comparisons was accepted at *p*<0.05 divided by the number of comparisons made, which was four *i.e. p*<0.0125. *p*-values shown in **bold** highlight significant differences.

### 3.3 Respiratory timing variability, apnoeas and sighs during normoxia in rats during quiet rest

**Fig. 1:**
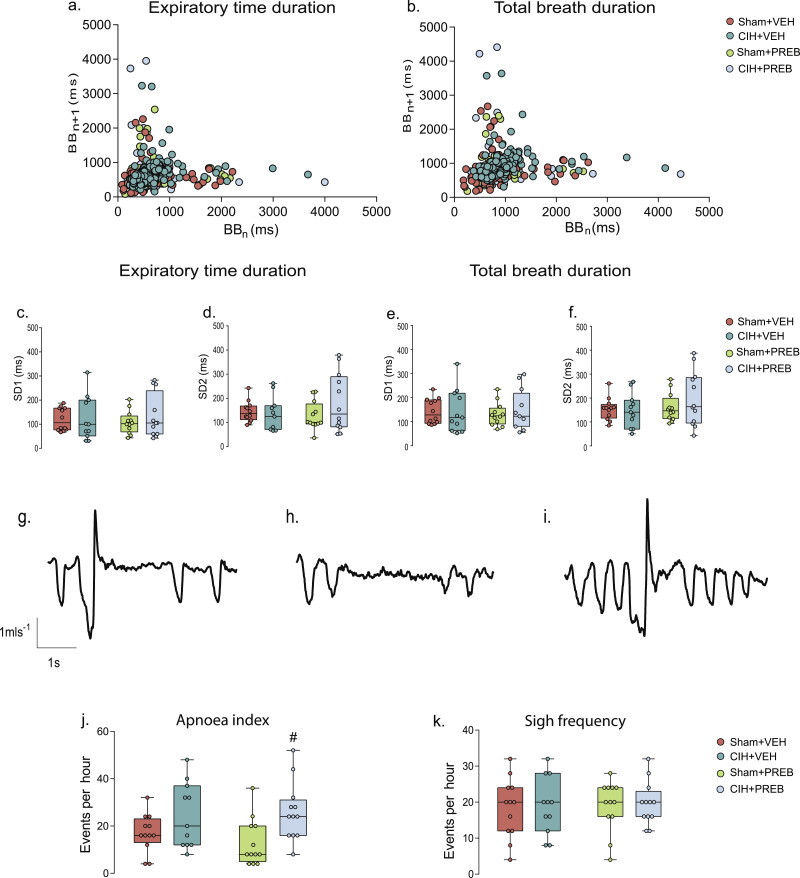
CIH increases apnoea index Poincaré plots of breath-to-breath (BBn) and subsequent breath-to-breath (BBn + 1) interval of expiratory duration (T_e_; a) and total breath duration (T_tot_; b) for Sham+VEH, CIH+VEH, Sham+PREB and CIH+PREB. Group data for T_e_ short-term variability (SD1; c) and long-term variability (SD2; d) and T_tot_ SD1 (e) and SD2 (f) in Sham+VEH, CIH+VEH, Sham+PREB and CIH+PREB rats during normoxia. Representative respiratory flow traces (downward deflections represent inspiration) illustrating a spontaneous sigh followed by an apnoea (g), a spontaneous apnoea (h) and a spontaneous sigh (i). Group data of apnoea index (j) and sigh frequency (k). CIH, chronic intermittent hypoxia; PREB, prebiotic; VEH, vehicle. Groups (c-f, j, k) are expressed as box and whisker plots (median, IQR and minimum to maximum values); n = 11–12. Groups were statistically compared using two-way ANOVA, followed by Fisher's least significant difference (LSD) *post hoc* where appropriate, or non-parametric Kruskal-Wallis test, followed by Mann-Whitney U test*,* where appropriate. Apnoea index was significantly affected by CIH exposure (*p* *=* 0.026; Fig. 1j). Assessments of respiratory timing variability and frequency of sighs were not different between groups (*p*>0.05; Fig. 1a-f, 1k). # *p* = 0.008, CIH+PREB *versus* Sham+PREB.

Assessments of short-term (SD1) and long-term (SD2) respiratory timing variability during normoxia did not reveal differences between groups (*p*>0.05; Fig. 1a-f). Apnoea index was significantly increased by CIH exposure (X^2^(3) = 9.284, *p =* 0.026, Fig. 1j), a consequence of alterations in spontaneous apnoea events; no statistically significant differences were evident in post-sigh apnoea events (Table 2). *Post hoc* analysis revealed that apnoea index was increased in CIH+PREB compared with Sham+PREB rats (*p =* 0.008; Fig. 1j). The frequency of sighs was not affected by CIH exposure or prebiotic administration (*p*>0.05; Fig. 1k). Sham+PREB had elevated sigh amplitude compared with Sham+VEH rats (Table 2). The major finding was that CIH exposure increased apnoea index during quiet breathing at rest (normoxia) and prebiotic administration did not prevent this aberrant phenotype.

### 3.4 Ventilatory and metabolic responsiveness to chemostimulation in rats during quiet rest

#### 3.4.1 Ventilatory and metabolic responsiveness to hypoxic chemostimulation

No significant differences were evident in CIH+VEH compared with Sham+VEH rats. CIH+PREB rats had decreased *f_R,_* V_I_, V_T_/T_i_ and increased T_i_ and T_e_ compared with Sham+PREB rats (Table 3). T_i_ was decreased in Sham+PREB compared with Sham+VEH rats (Table 3). Sigh frequency and amplitude were not different in CIH+VEH compared with Sham+VEH rats, but CIH+PREB rats had less frequent but larger sighs compared with CIH+VEH rats. Sigh frequency was reduced in CIH+PREB compared with Sham+PREB rats (Table 3). The major observation was that prebiotic administration reduced the frequency of sighs during hypoxia in CIH-exposed rats.

**Table 3:** Ventilatory and metabolic responsiveness to chemostimulation in rats during quiet rest.

	Sham+VEH	CIH+VEH	Sham+PREB	CIH+PREB	p-value (Kruskal-Wallis)	p-value (two-way ANOVA)	Sham+VEH v CIH+VEH	CIH+VEH v CIH+PREB	Sham+PREB v CIH+PREB	Sham+VEH v Sham+PREB
Hypoxia										
Δ *f_R_* (brpm)	58 ± 21	46 ± 18	64 ± 12	37 ± 13	N/A	Diet, *p* = 0.834; CIH, ***p*<0.0005**; Diet*CIH, *p* = 0.125	0.082	0.221	**<0.0005**	0.338
Δ V_I_ (ml/min/100 g)	46 ± 16	38 ± 10	50 ± 12	34 ± 14	N/A	Diet, *p* = 0.935; CIH, ***p*** **=** **0.003**; Diet*CIH, *p* = 0.344	0.140	0.544	**0.006**	0.462
Δ V_T_ (ml/100 g)	0.09 ± 0.10	0.10 ± 0.08	0.03 ± 0.07	0.08 ± 0.10	N/A	Diet, *p* = 0.093; CIH, *p* = 0.149; Diet*CIH, *p* = 0.584	–	–	–	–
Δ V_T_/T_i_ (ml/s/100 g)	1.6 ± 0.6	1.6 ± 0.6	2.4 ± 0.8	1.3 ± 1	N/A	Diet, *p* = 0.244; CIH, ***p*** **=** **0.021**; Diet*CIH, ***p*** **=** **0.022**	0.993	0.406	**0.001**	0.015
Δ T_i_ (ms)	−79 ± 36	−81 ± 27	−108 ± 27	−65 ± 24	**0.005**	N/A	0.786	0.295	**0.001**	**0.008**
Δ T_e_ (ms)	−228 ± 90	−170 ± 106	−230 ± 54	−125 ± 51	N/A	Diet, *p* = 0.365; CIH, ***p*** **=** **0.001**; Diet*CIH, *p* = 0.298	0.085	0.176	**0.002**	0.921
Δ VO_2_ (ml/min/100 g)	−0.7 ± 0.7	−0.4 ± 1	−1.3 ± 1	−1.5 ± 1	N/A	Diet, ***p*** **=** **0.003**; CIH, *p* = 0.858; Diet*CIH, *p* = 0.480	0.536	**0.011**	0.705	0.095
Δ VCO_2_ (ml/min/100 g)	0.3 ± 0.2	0.3 ± 0.3	0.3 ± 0.4	0.2 ± 0.2	0.412	N/A	–	–	–	–
Δ V_I_/VCO_2_	19 ± 8	14 ± 7	18 ± 8	12 ± 7	N/A	Diet, *p* = 0.487; CIH, ***p*** **=** **0.016**; Diet*CIH, *p* = 0.747	0.132	0.477	0.048	0.789
Sigh frequency (events per hr)	132 ± 46	155 ± 40	145 ± 29	112 ± 19	**0.008**	N/A	0.293	**0.002**	**0.009**	0.706
Sigh amplitude (ml/100g)	1.0 ± 0.14	1.1 ± 0.2	1.2 ± 0.23	1.4 ± 0.3	**0.001**	N/A	0.538	**0.001**	0.083	0.033
Hypercapnia										
Δ *f_R_ (brpm)*	60 ± 21	49 ± 17	57 ± 18	50 ± 21	N/A	Diet, *p* = 0.908; Exposure, *p* = 0.129; Diet*Exposure, *p* = 0.689	–	–	**–**	–
Δ V_I_(ml/min/100 g)	55 ± 18	55 ± 16	85 ± 29	73 ± 24	N/A	Diet, ***p*** **=** **0.001**; Exposure, *p* = 0.385; Diet*Exposure, *p* = 0.380	0.994	0.064	0.214	**0.002**
Δ V_T_ (ml/100 g)	0.18 ± 0.18	0.2 ± 0.1	0.23 ± 0.1	0.3 ± 0.1	**0.019**	N/A	0.268	0.056	0.729	0.021
Δ V_T_/T_i_ (ml/s/100 g)	1.4 ± 0.7	1.6 ± 0.7	2.8 ± 1.1	2.5 ± 0.7	N/A	Diet, ***p*<0.0005**; Exposure, *p* = 0.979; Diet*Exposure, *p* = 0.327	0.480	**0.01**	0.494	**<0.0005**
Δ T_i_ (ms)	−53 ± 28	−59 ± 19	−72 ± 27	67 ± 24	0.307	N/A	–	–	–	–
Δ T_e_ (ms)	−304 ± 109	−232 ± 89	−223 ± 67	−190 ± 112	0.081	N/A	–	–	–	–
Δ VCO_2_ (ml/min/100 g)	0.4 ± 0.7	0.2 ± 0.6	0.7 ± 0.6	0.3 ± 0.9	0.213	N/A	–	–	–	–
Δ V_I_/VCO_2_	27 ± 16	41 ± 14	28 ± 19	41 ± 33	0.150	N/A	–	–	–	–
Sigh frequency (events per hr)	10 ± 5	17 ± 6	23 ± 21	20 ± 6	**0.001**	N/A	**0.009**	0.333	0.333	**0.001**
Sigh amplitude (ml/100 g)	1.1 ± 0.3	1.5 ± 0.3	1.6 ± 0.3	1.4 ± 0.2	**0.017**	N/A	0.031	0.389	0.389	**0.006**
Hypoxic hypercapnia										
Δ *f_R_ (brpm)*	58 ± 10	50 ± 16	65 ± 18	56 ± 11	N/A	Diet, *p* = 0.157; Exposure, *p* = 0.062; Diet*Exposure, *p* = 0.790	–	–	**–**	–
Δ V_I_ (ml/min/100 g)	76 ± 20	69 ± 15	110 ± 20	95 ± 23	N/A	Diet, ***p*<0.0005**; Exposure, *p* = 0.090; Diet*Exposure, *p* = 0.562	0.446	**0.006**	0.088	**<0.0005**
Δ V_T_ (ml/100 g)	0.3 ± 0.2	0.3 ± 0.1	0.4 ± 0.2	0.4 ± 0.2	**0.030**	N/A	0.683	0.286	0.094	**0.010**
Δ V_T_/T_i_ (ml/s/100 g)	2.0 ± 0.9	2.0 ± 0.7	3.4 ± 0.8	2.8 ± 1.0	N/A	Diet, ***p*<0.0005**; Exposure, *p* = 0.465; Diet*Exposure, *p* = 0.204	0.713	0.070	0.135	**0.001**
Δ T_i_ (ms)	−57 ± 33	−73 ± 17	−76 ± 36	−74 ± 22	N/A	Diet, *p* = 0.252; Exposure, *p* = 0.412; Diet*Exposure, *p* = 0.328	–	–	–	–
Δ T_e_ (ms)	−291 ± 194	−234 ± 112	−301 ± 96	−271 ± 54	0.450	N/A	–	–	–	–
Δ VCO_2_ (ml/min/100 g)	−0.3 ± 0.4	−0.3 ± 0.4	0.2 ± 0.4	−0.1 ± 0.6	N/A	**Diet, *p*** **=** **0.039**; Exposure, *p* = 0.304; Diet*Exposure, *p* = 0.322	0.980	0.435	0.132	0.030
Δ V_I_/VCO_2_	62 ± 23	55 2 ± 5	55 ± 21	53 ± 26	N/A	Diet, *p* = 0.573; `Exposure, *p* = 0.586; Diet*Exposure, *p* = 0.762	–	–	–	–

*f_R_,* respiratory frequency (brpm, breaths per minute); V_I_, minute ventilation; *V*_T,_ tidal volume; *V*_T_/T_i_, mean inspiratory flow; T_i_, inspiratory time; T_e_, expiratory time; *V*O_2_, oxygen consumption; *V*CO_2,_ carbon dioxide production; V_I_/VCO_2,_ ventilatory equivalent; CIH, chronic intermittent hypoxia; PREB, prebiotic; VEH, vehicle. Data are shown as mean ± SD and were statistically compared using two-way ANOVA, followed by Fisher's least significant difference (LSD) *post hoc* where appropriate, or non-parametric Kruskal-Wallis test, followed by Mann-Whitney U test*,* where appropriate. Statistical significance for multiple comparisons was accepted at *p*<0.05 divided by the number of comparisons made, which was four *i.e. p*<0.0125. *p*-values shown in **bold** highlight significant differences.

#### 3.4.2 Ventilatory and metabolic responsiveness to hypercapnic chemostimulation

CIH exposure elevated sigh frequency during hypercapnia, as such CIH+VEH rats had increased generation of sigh compared with Sham+VEH rats. Other respiratory and metabolic parameters were not different in CIH+VEH compared with Sham+VEH rats in response to hypercapnia (Table 3). Prebiotic administration in CIH-exposed rats elevated V_T_/T_i_ compared with CIH+VEH rats. Interestingly, Sham+PREB rats had elevated ventilation (V_I_) and increased drive to breathe (V_T_/T_i_) in response to hypercapnia compared with Sham+VEH rats, with no change in V_I_/VCO_2_ (Table 3). Furthermore, Sham+PREB rats had augmented sigh frequency and amplitude compared with Sham+VEH rats. There was no difference between CIH+PREB and Sham+PREB rats (Table 3). The major finding was that prebiotic fibre supplementation increased sigh frequency and the ventilatory response to hypercapnia.

#### 3.4.3 Ventilatory and metabolic responsiveness to hypoxic hypercapnic chemostimulation

No significant differences were evident in CIH+VEH compared with Sham+VEH rats. CIH+PREB rats had elevated V_I_ compared with CIH+VEH rats. Sham+PREB rats had an elevated ventilatory response to hypoxic hypercapnia compared with Sham+VEH rats, evident by increased V_I_, V_T_ and V_T_/T_I_; V_I_/VCO_2_ was not different between groups (Table 3). Furthermore, there was no apparent difference between Sham+PREB and CIH+PREB rats. The major observation was that prebiotic administration elevated the ventilatory response to hypoxic hypercapnia.

### 3.5 Baseline cardiorespiratory and blood gas parameters in anaesthetised rats

**Fig. 2:**
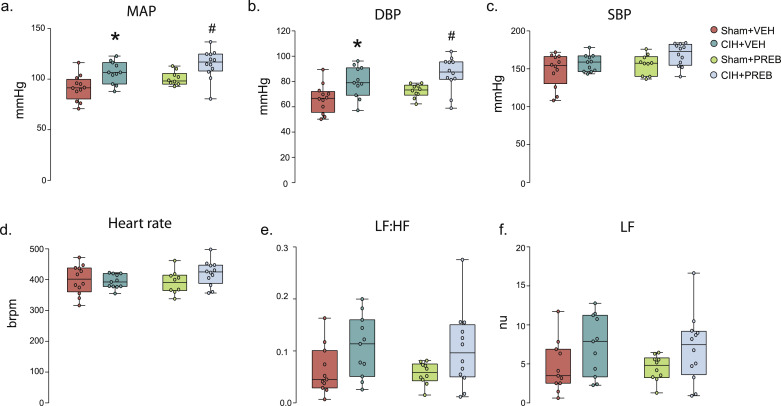
CIH causes hypertension and cardiac autonomic imbalance Group data for MAP (a), DBP (b), SBP (C), heart rate (D), LF: HF (e) and LF (f) for Sham+VEH, CIH+VEH, Sham+PREB and CIH+PREB. MAP, mean arterial blood pressure; DBP, diastolic blood pressure; SBP, systolic blood pressure; LF, low-frequency band; HF, high frequency band; CIH, chronic intermittent hypoxia; PREB, prebiotic; VEH, vehicle. Groups (a-f) are expressed as box and whisker plots (median, IQR and minimum to maximum values); n = 10–12 for all groups. Groups were statistically compared using two-way ANOVA, followed by Fisher's least significant difference (LSD) *post hoc* where appropriate, or non-parametric Kruskal-Wallis test, followed by Mann-Whitney U test*,* where appropriate**.** CIH significantly affected MAP, DBP, LF: HF and LF (*p*<0.005, *p<*0.0005, *p* *=* 0.008 and *p* *=* 0.017, respectively; Fig. 2a, 2b, 2e, 2f). There was no change in SBP or HR (*p*>0.05, Fig. 2c, 2d).* *p* = 0.004, CIH+VEH *versus* Sham+VEH; # *p*<0.01, CIH+PREB *versus* Sham+PREB.

CIH exposure had no effect on respiration in the anaesthetised rat during baseline conditions (Table 4). V_I_ and V_T_ were increased in PREB+CIH rats compared with CIH-exposed rats (Table 4). CIH exposure significantly increased diastolic blood pressure (DBP) (CIH; F (1,41) = 16.321, *p<*0.0005, ƞ^2^=0.285, Fig. 2b). As a consequence, mean arterial blood pressure (MAP) was elevated (CIH; F (1, 41) = 17.485, *p*<0.005, ƞ^2^=0.299). *Post hoc* analysis revealed CIH+VEH had elevated blood pressure compared with Sham+VEH rats (DBP, *p =* 0.006, Fig. 2b; MAP, *p* = 0.004, Fig. 2a). DBP was not restored by prebiotic administration as CIH+PREB had elevated DBP compared with CIH+VEH rats (*p =* 0.007, Fig. 2b). There was no statistical difference evident in MAP between CIH+PREB compared with CIH+VEH (*p*>0.05, Fig. 2a), and MAP was elevated in CIH+PREB compared with Sham+PREB rats (*p =* 0.006; Fig. 2a). CIH exposure or prebiotic administration had no effect on systolic blood pressure or heart rate (*p*>0.05, Fig. 2c, 2d). CIH exposure had no effect on haematocrit and haemoglobin concentrations; Sham+PREB had reduced concentrations compared with Sham+VEH (Table 4).

**Table 4:** Baseline ventilation, blood gases and cardiovascular parameters in anaesthetised rats.

	Sham+VEH	CIH+VEH	Sham+PREB	CIH+PREB	p-value (Kruskal-Wallis)	p-value (two-way ANOVA)	Sham+VEH v CIH+VEH	CIH+VEH v CIH+PREB	Sham+PREB v CIH+PREB	Sham+VEH v Sham+PREB
f_R_ (brpm)	99 ± 11	99 ± 11	98 ± 10	93 ± 5	N/A	Diet, *p* = 0.448; CIH, *p* = 0.376; Diet*CIH, *p* = 0.397	–	–	–	–
V_I_ (ml/ min/ 100 g)	24 ± 5	21 ± 3	25 ± 3	27 ± 3	N/A	Diet, ***p*** **=** **0.003**; CIH, *p* = 0.642; Diet*CIH, *p* = 0.088	0.111	**0.001**	0.387	0.339
V_T_ (ml/ 100 g)	0.25 ± 0.05	0.22 ± 0.03	0.26 ± 0.02	0.29 ± 0.03	N/A	Diet, ***p*<0.0005**; CIH, *p* = 0.998; Diet*CIH, ***p*** **=** **0.019**	0.081	**<0.0005**	0.103	0.306
ETCO_2_	5 ± 1.6	6 ± 0.5	5.7 ± 0.6	6 ± 0.9	N/A	Diet, *p* = 0.274; CIH, *p* = 0.448; Diet*CIH, *p* = 0.316	–	–	–	–
pH	7.37 ± 0.04	7.33 ± 0.02	7.34 ± 0.02	7.34 ± 0.03	N/A	Diet, *p* = 0.427; CIH, ***p*** **=** **0.049**; Diet*CIH, *p* = 0.211	0.023	0.738	0.598	0.155
PaCO_2_ (mmHg)	46 ± 6.4	51 ± 3.7	51 ± 4.4	49 ± 4.9	N/A	Diet, *p* = 0.394; CIH, *p* = 0.278; Diet*CIH, ***p*** **=** **0.049**	0.03	0.406	0.518	0.050
PaO_2_ (mmHg)	97 ± 3.8	99 ± 6.9	97 ± 13	98 ± 6.2	N/A	Diet, *p* = 0.416; CIH, *p* = 0.863; Diet*CIH, *p* = 0.822	–	–	–	–
Haematocrit (%)	49 ± 2	49 ± 2.8	45 ± 2.8	42 ± 13	**0.004**	N/A	0.975	0.035	0.947	**0.004**
[Hb] (g/dl)	16.8 ± 0.7	16.8 ± 1.0	15.4 ± 1.0	15.7 ± 1.3	N/A	Diet, ***p*<0.0005**; CIH, *p* = 0.696; Diet*CIH, *p* = 0.580	0.907	0.013	0.510	**0.002**

*f*_R_*,* respiratory frequency (brpm, breaths per minute); *V*_I_, minute ventilation; *V*_T,_ tidal volume; ETCO_2,_ end-tidal carbon dioxide production; Pco_2_, partial pressure of arterial carbon dioxide_;_ Pao_2_, partial pressure of arterial oxygen; [Hb], haemoglobin concentration; CIH, chronic intermittent hypoxia; PREB, prebiotic; VEH, vehicle. Data are shown as mean ± SD and were statistically compared using two-way ANOVA, followed by Fisher's least significant difference (LSD) *post hoc* where appropriate, or non-parametric Kruskal-Wallis test, followed by Mann-Whitney U test*,* where appropriate. Statistical significance for multiple comparisons was accepted at *p*<0.05 divided by the number of comparisons made, which was four *i.e. p*<0.0125. *p*-values shown in **bold** highlight significant differences.

CIH exposure increased the low-frequency band (LF) (CIH; nu, F (1, 40) = 6.170, *p =* 0.017, ƞ^2^=0.134, Fig. 2f;%, F (1, 40) = 6.723, *p =* 0.013, ƞ^2^=0.144, Table 5) and decreased the high-frequency band (HF; nµ) (CIH, F (1, 40) = 1.159, *p =* 0.014, ƞ^2^=0.142, Table 5) elevating the LF: HF ratio (CIH, F (1, 40) = 7.748, *p =* 0.008, ƞ^2^=0.162, Fig. 2e) during steady-state baseline recordings, indicating sympathetic dominance. LF:HF was increased in CIH+VEH compared with Sham+VEH rats (*p =* 0.059, Fig. 2e). There was no difference in CIH+PREB compared with CIH+VEH rats, however CIH+PREB rats had elevated LF:HF ratio compared with Sham+PREB rats (*p =* 0.053, Fig. 2e). After adjusting for multiple comparisons these changes were not statistically significant (Table 5). Other heart rate variability parameters were not different between groups (Table 5). The major finding was that CIH exposure caused hypertension and cardiac autonomic imbalance, which were not alleviated by prebiotic supplementation.

**Table 5:** Heart rate variability in the urethane anaesthetised rats.

	Sham+VEH	CIH+VEH	Sham+PREB	CIH+PREB	p-value (Kruskal-Wallis)	p-value (two-way ANOVA)	Sham+VEH v CIH+VEH	CIH+VEH v CIH+PREB	Sham+PREB v CIH+PREB	Sham+VEH v Sham+PREB
Total Power (ms^2^)	16 ± 28	10 ± 11	4 ± 3	13 ± 15	0.653	N/A	–	–	–	–
HF (nµ)	78 ± 11	71 ± 10	84 ± 7	73 ± 10	N/A	Diet, *p* = 0.288; CIH, ***p*** **=** **0.014**; Diet*CIH, *p* = 0.662	0.138	0.648	0.039	0.301
VLF (ms^2^)	0.4 ± 0.3	1.0 ± 1.7	0.4 ± 0.2	0.3 ± 0.5	0.163	N/A	–	–	–	–
LF (ms^2^)	0.3 ± 0.5	0.4 ± 0.5	0.1 ± 0.1	0.5 ± 0.7	0.214	N/A	–	–	–	–
HF (ms^2^)	12 ± 20	6 ± 7	3 ± 2	9 ± 12	0.590	N/A	–	–	–	–
VFL (%)	15 ± 14	24 ± 26	16 ± 11	9 ± 11	0.251	N/A	–	–	–	–
LF (%)	4 ± 2	5 ± 3	4 ± 1	6 ± 4	N/A	Diet, *p* = 0.658; CIH, ***p*** **=** **0.013**; Diet*CIH, *p* = 0.619	0.146	0.497	0.035	0.970
HF (%)	66 ± 15	53 ± 15	70 ± 11	67 ± 11	0.109	N/A	–	–	–	–
Average RRI (ms)	153 ± 18	152 ± 9	155 ± 14	144 ± 14	N/A	Diet, *p* = 0.445; CIH, *p* = 0.189; Diet*CIH, *p* = 0.238	–	–	–	–
Median RRI (ms)	153 ± 19	152 ± 10	155 ± 14	144 ± 14	N/A	Diet, *p* = 0.460; CIH, *p* = 0.199; Diet*CIH, *p* = 0.272	–	–	–	–
SDRR (ms)	3.3 ± 2.5	3 ± 2	2 ± 0.5	3.6 ± 2.3	0.626	N/A	–	–	–	–
CVRR	0.02 ± 0.02	0.02 ± 0.01	0.01 ± 0.003	0.03 ± 0.02	0.373	N/A	–	–	–	–
SD Rate (bpm)	8.2 ± 5.8	7.8 ± 5.2	5.2 ± 1.5	11 ± 8.4	0.380	N/A	–	–	–	–
SDSD (ms)	4.5 4.7	4.1 ± 3.8	2.6 ± 1.2	5.5 ± 4.2	0.586	N/A	–	–	–	–
RMSSD (ms)	4.5 ± 4.8	4.1 ± 3.8	2.6 ± 1.2	5.5 ± 4.2	0.586	N/A	–	–	–	–
SD1 (ms)	3.2 ± 3.3	2.9 ± 2.7	1.8 ± 0.8	3.9 ± 2.9	0.586	N/A	–	–	–	–
SD2 (ms)	3.1 ± 1.9	2.8 ± 1.5	2.2 ± 0.5	3.2 ± 1.6	N/A	Diet, *p* = 0.489; CIH, *p* = 0.373; Diet*CIH, *p* = 0.131	–	–	–	–

VLF, very low frequency; LF, low frequency; HF, high frequency; RRI, R-R interval; SDRR, standard deviation of R-R interval; CVRR, coefficient of variance of R-R intervals; SD rate, standard deviation of heart rate; SDSD, standard deviation of successive R-R intervals; RMSSD, root mean square of successive R-R interval differences; SD1, short term variability; SD2, long-term variability; CIH, chronic intermittent hypoxia; PREB, prebiotic; VEH, vehicle. Data are shown as mean ± SD and were statistically compared using two-way ANOVA, followed by Fisher's least significant difference (LSD) *post hoc* where appropriate, or non-parametric Kruskal-Wallis test, followed by Mann-Whitney U test*,* where appropriate. Statistical significance for multiple comparisons was accepted at *p*<0.05 divided by the number of comparisons made, which was four *i.e. p*<0.0125. *p*-values shown in **bold** highlight significant differences.

### 3.6 Cardiorespiratory responses to 5-HT_3_ receptor agonism evoking the cardiopulmonary reflex

**Fig. 3:**
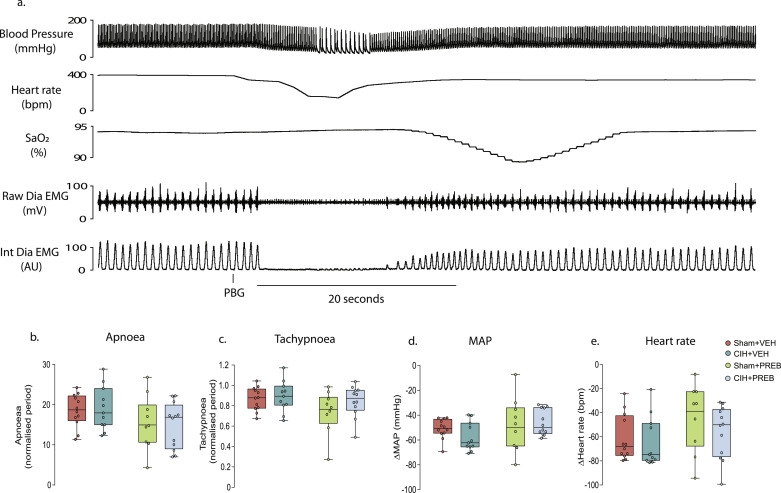
CIH did not alter cardiorespiratory responses to pulmonary vagal afferent C-fibre stimulation a) Representative traces of blood pressure, heart rate, peripheral oxygen saturation (SpO_2_) and raw and integrated diaphragm (Dia) electromyogram (EMG) activity during intravenous administration of the 5-HT_3_ agonist, phenylbiguanide (25 μg.kg^−1^ i.v.). Group data for maximum apnoea duration (b) and tachypnoea (c) normalised to respective baseline respiratory period in Sham+VEH, CIH+VEH, Sham+PREB and CIH+PREB rats. Absolute change in MAP (d) and heart rate (e) in response to PBG in Sham, CIH, Sham+PREB and CIH+PREB rats. MAP, mean arterial blood pressure; CIH, chronic intermittent hypoxia; PREB, prebiotic; VEH, vehicle. Data (b-e) are expressed as box and whisker plots (median, IQR and minimum to maximum values); *n* = 9–12. Groups (b-e) were statistically compared using two-way ANOVA, followed by Fisher's least significant difference (LSD) *post hoc* where appropriate, or non-parametric Kruskal-Wallis test, followed by Mann-Whitney U test*,* where appropriate. CIH exposure had no effect on hypotension, bradycardia, apnoea or post-apnoea induced tachypnoea (*p*>0.05; Fig. 3b-e). Prebiotic supplementation had a significant effect on apnoea duration (*p* *=* 0.032; Fig. 3b), with no effect on any of the other parameters (*p*<0.05; Fig. 3c-e).

Stimulation of 5-HT_3_ receptors expressed on pulmonary vagal afferent nerve fibres, using PBG, evoked the integrated cardiopulmonary reflex. CIH exposure had no effect on hypotension, bradycardia, apnoea or post-apnoea induced tachypnoea associated with the pulmonary chemoreflex (Fig. 3b-e). Prebiotic supplementation altered apnoea duration (Diet, F (1, 41) = 4.950, *p =* 0.032, ƞ^2^=0.108, Fig. 3b)*,* however, *post hoc* analysis revealed no differences between groups. There was no significant difference between groups in all other parameters (Fig. 3c-e). The major finding was that pulmonary chemoreflex responses to vagal afferent stimulation were unaffected by CIH exposure.

### 3.7 Cardiovascular responses to pharmacological blockade of sympathetic activity in anaesthetised rats

The blood pressure response to β1 adrenoceptor antagonism (atenolol) was significantly increased by exposure (X^2^(3) = 9.347, *p =* 0.025, Table 6). CIH+VEH was not different compared with Sham+VEH rats. There was a greater depressor response in CIH+PREB compared with Sham+PREB rats; the associated bradycardia was similar between all groups (Table 6). Intravenous infusion of the non-selective β-adrenoceptor blocker (propranolol), and sympathetic ganglion blocker (hexamethonium) evoked similar bradycardia and hypotensive responses across all groups (Table 6).

**Table 6:** Cardiovascular responses to pharmacological blockade of sympathetic activity in urethane anaesthetised rats.

	Sham+VEH	CIH+VEH	Sham+PREB	CIH+PREB	p-value (Kruskal-Wallis)	p-value (two-way ANOVA)	Sham+VEH v CIH+VEH	CIH+VEH v CIH+PREB	Sham+PREB v CIH+PREB	Sham+VEH v Sham+PREB
Propranolol (% change from baseline)										
SBP	−24 ± 9	−22 ± 6	−21 ± 6	−23 ± 5	0.906	N/A	–	–	–	–
HR	−12 ± 5	−18 ± 13	−16 ± 6	−19 ± 11	0.092	N/A	–	–	–	–
Atenolol (% change from baseline)										
SBP	8 ± 10	9 ± 5	3 ± 5	7 ± 3	**0.02**5	N/A	0.268	0.538	**0.006**	0.235
HR	−27 ± 17	−28 ± 11	−28 ± 10	−30 ± 5	0.276	N/A	–	–	–	–
Hexamethonium (% change from baseline)										
SBP	−48 ± 9	−46 ± 8	−41 ± 7	−46 ± 6	N/A	Diet, *p* = 0.205; CIH, *p* = 0.422; Diet*CIH, *p* = 0.184	–	–	–	–
HR	−14 ± 17	−15 ± 18	−13 ± 14	−12 ± 11	0.743	N/A	–	–	–	–

SBP, systolic blood pressure; HR, heart rate; CIH, chronic intermittent hypoxia; PREB, prebiotic; VEH, vehicle. Data are shown as mean ± SD and were statistically compared using two-way ANOVA, followed by Fisher's least significant difference (LSD) *post hoc* where appropriate, or non-parametric Kruskal-Wallis test, followed by Mann-Whitney U test*,* where appropriate. Statistical significance for multiple comparisons was accepted at *p*<0.05 divided by the number of comparisons made, which was four *i.e. p*<0.0125. *p*-values shown in **bold** highlight significant differences.

### 3.8 Pons and medulla oblongata neurochemistry

**Fig. 4:**
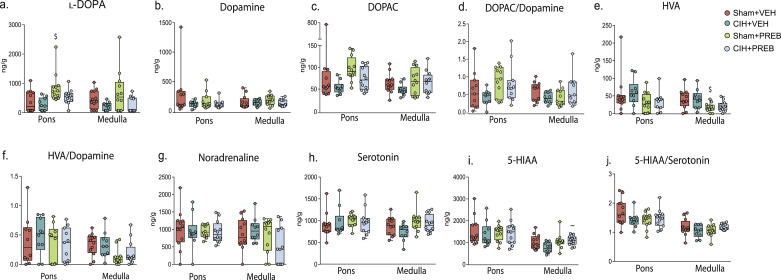
Prebiotic administration alters brainstem neurochemistry Group data for _l_-DOPA (a), dopamine (b), DOPAC (c) DOPAC/Dopamine (d), homovanillic acid (e), homovanillic acid/dopamine ratio (f), noradrenaline (g), serotonin (h), 5-HIAA (i) and 5-HIAA/Serotonin ratio (j) in Sham+VEH, CIH+VEH, Sham+PREB and CIH+PREB. _l_-DOPA, _l_-3,4-dihydroxyphenylalanine; DOPAC, 3,4-dihydroxyphenylacetic acid; 5-HIAA, 5-hydroindoleacetic acid; CIH, chronic intermittent hypoxia; PREB; prebiotic; VEH, vehicle. Data (a-j) are expressed as box and whisker plots (median, IQR and minimum to maximum values); *n* = 10–12. Groups were statistically compared using two-way ANOVA, followed by Fisher's least significant difference (LSD) *post hoc* where appropriate, or non-parametric Kruskal-Wallis test, followed by Mann-Whitney U test*,* where appropriate. _l_-DOPA (*p* *=* 0.003; Fig. 4a) and DOPAC (*p* *=* 0.006; Fig. 4c) concentrations in the pontine region as well as DOPAC/DA (Diet*CIH, *p* *=* 0.042; Fig. 4d), HVA (*p* *=* 0.001; Fig. 4e), HVA/DA (*p* *=* 0.020; Fig. 4f), 5-HT (Diet, *p* *=* 0.043; Fig. 4h) and 5-HIAA (*p* *=* 0.043; Fig. 4i) concentrations in the medulla oblongata are different. Other monoamine, metabolites and precursors were not statistically different between groups (*p*>0.05; Fig. 4b, 4g, 4h, 4j). ~ *p* *=* 0.008, CIH+PREB *versus* CIH+VEH; $ *p* *=* 0.006, Sham+PREB *versus* Sham+VEH.

Comparison of _l_-DOPA and DOPAC concentrations in the pons, as well as DOPAC/DA, HVA, HVA/DA, 5-HT and 5-HIAA concentrations in the medulla oblongata revealed group differences (*p*<0.05; Fig. 4a, 4c-f, 4h, 4i). However, *post hoc* analysis revealed that monoamine, monoamine metabolites and precursors were not different in CIH+VEH compared with Sham+VEH rats in the pons or medulla oblongata (Fig. 4a-j). Pontine _l_-DOPA (*p* = 0.020) and medulla oblongata 5-HIAA (*p* = 0.008) concentrations were significantly increased, with medulla oblongata HVA (*p* = 0.041) levels decreased in CIH+PREB compared with CIH+VEH rats. CIH+PREB rats had reduced pontine _l_-DOPA (*p* = 0.038) concentrations compared with Sham+PREB rats. Sham+PREB rats had increased pontine DOPAC (*p* = 0.021) as well as reduced medulla oblongata HVA (*p* = 0.006) and HVA/DA (*p* = 0.016) concentrations compared with Sham+VEH rats. Additionally, Sham+PREB rats had elevated pontine _l_-DOPA (*p* = 0.012) concentrations compared with Sham+VEH rats. After adjusting for multiple comparisons, differences in pontine _l_-DOPA and medulla oblongata HVA concentrations in Sham+PREB compared with Sham+VEH and medulla oblongata 5-HIAA concentrations in CIH+PREB compared with Sham+PREB remained significantly different. In summary, prebiotic administration, but not CIH, altered brainstem neurochemistry.

### 3.9 Plasma cytokine and corticosterone concentrations

**Fig. 5:**
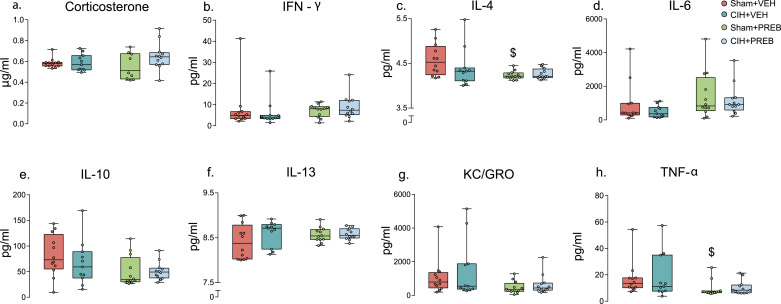
Corticosterone and inflammatory mediators were equivalent between groups Group data for corticosterone concentration (a), IFN-γ (b), IL-4 (c), IL-6 (d), IL-10 (e), IL-13 (f), KC/GRO (g) and TNF-α (h) in Sham+VEH, CIH+VEH, Sham+PREB and CIH+PREB. IFN-γ, interferon-γ; IL-4, interleukin-4; IL-6, interleukin-6; IL-10, interleukin-10;  IL-13, interleukin-13;   TNF-α, tumour necrosis factor-α; KC/GRO, keratinocyte chemoattractant/growth-related oncogene; CIH, chronic intermittent hypoxia; PREB; prebiotic; VEH, vehicle. Data (a-h) are expressed as box and whisker plots (median, IQR and minimum to maximum values); *n* = 11–12. Groups (a-h) were statistically compared using two-way ANOVA, followed by Fisher's least significant difference (LSD) *post hoc* where appropriate, or non-parametric Kruskal-Wallis test, followed by Mann-Whitney U test*,* where appropriate. Pro-inflammatory cytokines, IL-4 (*p* *=* 0.045; Fig. 5c) and TNF-α (*p* *=* 0.013; Fig. 5h) were affected by prebiotic administration. $ *p* = 0.003, Sham+PREB *versus* Sham+VEH.

Pro-inflammatory cytokines, IL-4 (X^2^(3) = 8.042, *p =* 0.045, Fig. 5c) and TNF-α (X^2^(3) = 10.784, *p =* 0.013, Fig. 5h) were different between groups. *Post hoc* analysis adjusted for multiple comparisons revealed that CIH exposure had no significant effect on IL-4 or TNF-α levels. However, TNF-α and IL-4 were decreased in Sham+PREB compared with Sham+VEH rats. All other pro-inflammatory cytokines and corticosterone concentrations were not different between groups (Fig. 5a-b, 5d-g). In summary, CIH exposure had no effect on plasma cytokine or corticosterone concentrations. Prebiotic administration reduced TNF-α and IL-4 concentrations compared with VEH rats.

### 3.10 Caecal microbiota

#### 3.10.1 Microbiota composition and diversity

**Fig. 6:**
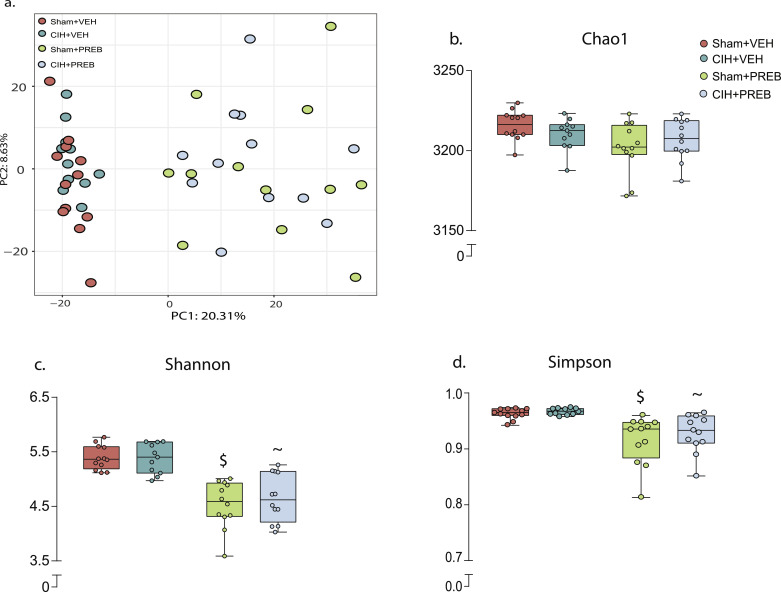
Prebiotic administration alters rat caecal microbiota structure Group data for principal coordinate analysis (a) in 2-dimensional representations, Chao1 (b), Shannon (c), Simpson (d) in Sham+VEH, CIH+VEH, Sham+PREB and CIH+PREB. CIH, chronic intermittent hypoxia; PREB; prebiotic; VEH, vehicle. Data (b-e) are expressed as box and whisker plots (median, IQR and minimum to maximum values); *n* = 11–12. Data (b-e) were statistically compared by non-parametric Mann-Whitney *U* test. P-value adjusted; ~ *p*<0.01, CIH+PREB *versus* CIH+VEH; $ *p* <0.0001, Sham+PREB *versus* Sham+VEH;.

Principal component analysis revealed that CIH exposure did not affect β-diversity of caecal contents. Prebiotic administration shifted β-diversity in Sham+PREB and CIH+PREB compared with Sham+VEH and CIH+VEH rats, respectively (Fig. 6a, *p* = 0.001, PERMANOVA). CIH exposure had no effect on indices of alpha diversity (Fig. 6b-d). Prebiotic treatment significantly reduced bacteria species evenness in all statistical comparisons, indicated by decreases in Shannon and Simpson indices of alpha diversity (Fig. 6c-d). However, bacterial species richness, indicated by Chao1 index was not affected by prebiotic administration (Fig. 6b). These findings suggest changes in a select number of bacterial species with no overall difference in bacterial richness of the caecum.

**Fig. 7:**
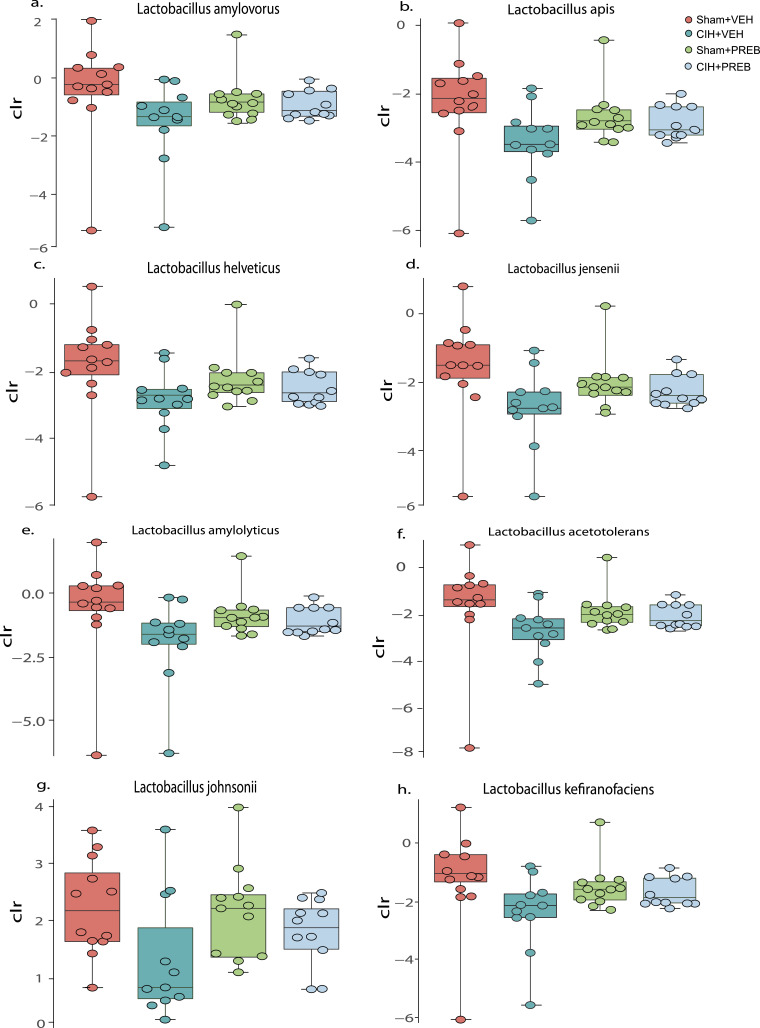
*Lactobacilli* species are decreased in CIH+VEH compared with Sham+VEH Group data for *Lactobacillus amylovorous* (a), *Lactobacillus apis* (b), *Lactobacillus helveticus* (c), *Lactobacillus jensenii* (d), *Lactobacillus amyloyticus* (e), *Lactobacillus acetotolers* (f), *Lactobacillus johnsonii* (g) and *Lactobacillus kefiranofaciens* (h) in Sham+VEH, CIH+VEH, Sham+PREB and CIH+PREB. CIH, chronic intermittent hypoxia; PREB; prebiotic; VEH, vehicle. Data (a-h) are expressed as box and whisker plots (median, IQR and minimum to maximum values); *n* = 11–12. Data (b-e) were statistically compared by non-parametric Mann-Whitney *U* test.

BH adjustment for multiple comparisons at bacterial species level did not reveal statistically significant differences between CIH+VEH and Sham+VEH rats given the exhaustive multiple comparisons performed. However, large effect sizes were evident in the relative abundance of *Lactobacillus* species between CIH+VEH and Sham+VEH rats (Fig. 7a-h). Similarly, no statistically significant difference was evident between Sham+PREB and CIH+PREB rats. The relative abundance of multiple bacterial species were different in CIH+VEH rats compared with CIH+PREB rats and in Sham+VEH rats compared with Sham+PREB rats. The largest difference between these comparisons was due to a significant increase in the beneficial bacterial species *Bifidobacterium animalis* in the prebiotic groups (Supplementary excel).

#### 3.10.2 Gut-brain module and gut-metabolic module analysis

**Fig. 8:**
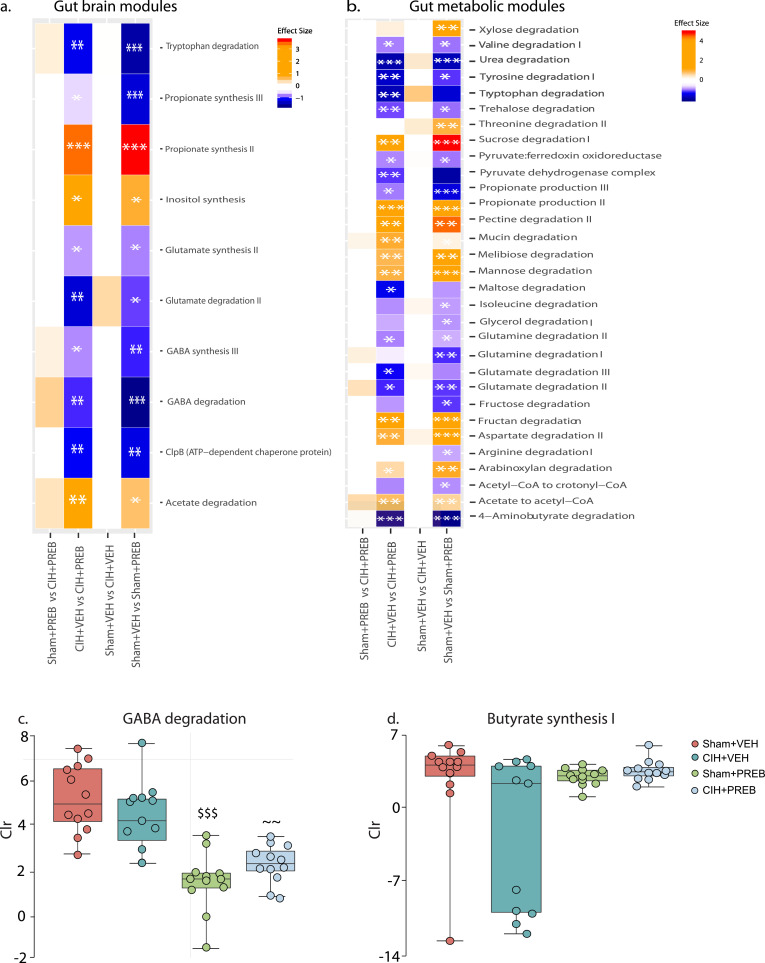
Prebiotic administration alters GBMs and GMMs Group data for GBMs (a) and GMMs (b) in heatmap representation, GABA degradation (c) and Butyrate synthesis I (d) in Sham+VEH, CIH+VEH, Sham+PREB and CIH+PREB. CIH, chronic intermittent hypoxia; PREB; prebiotic; VEH, vehicle. Data (c-d) are expressed as box and whisker plots (median, IQR and minimum to maximum values); *n* = 11–12. A pairwise implementation of the *aldex.ttest()* function was used to compare multiple groups. CIH exposure did not alter GBMs and GMMs. Prebiotic administration significantly modulated many metagenomes of the GBMs and GMMs. A positive effect size indicates an increase in prebiotic treated rats, a negative effect size indicates a decrease in prebiotic treated rats. Benjamini-Hochberg corrected q-values,* *q*<0.05, ** *q*<0.01, *** *q*<0.001. GABA degradation and Butyrate synthesis I diverge in CIH-exposed compared with Sham rats, depending on prebiotic administration. ~ *p*<0.01, CIH+PREB *versus* CIH+VEH; $$$ *p*< 0.001, Sham+PREB *versus* Sham+VEH.

Using known GBMs and GMMs we evaluated gut microbial functions. Our novel findings reveal that CIH exposure did not affect GBMs and GMMs analysis of caecal microbiota. However, other unidentified pathways might be affected. Prebiotic administration altered the microbial potential of 10 and 31 GBMs and GMMs, respectively (adjusted *p*<0.05; Fig. 8a,b). Several GMMs and GBMs were enriched (positive effect size) and reduced (negative effect size) in prebiotic treated rats compared with vehicle treated rats. Interestingly, GABA degradation (*p* = 0.9; effect size=0.4, CIH+VEH *versus* Sham+VEH; *p* = 0.9, effect size=0.4, CIH+PREB *versus* Sham+PREB; *p* = 0.009, effect size ~1, CIH+PREB *versus* CIH+VEH; *p* = 0.001, effect size ~1, Sham+PREB *versus* Sham+VEH; Fig. 8c) and butyrate synthesis I (*p* = 0.9; effect size=0.4, CIH+VEH *versus* Sham+VEH; *p* = 0.9, effect size=0.3, CIH+PREB *versus* Sham+PREB; *p* = 0.2, effect size=0.6, CIH+PREB *versus* CIH+VEH; *p* = 0.9, effect size=0.4, Sham+PREB *versus* Sham+VEH; Fig. 8d) abundance trended in diverging directions in CIH-exposed rats compared with Sham rats, depending on prebiotic or vehicle administration.

### 3.11 Correlation analysis

Hierarchical All-against-all correlation analysis showed that the relative abundance of *Francisella* sp. *FSC1006* strongly negatively correlated with sigh frequency during hypercapnia in rats that did not receive prebiotics. No other significant correlations were evident when bacterial species were assessed against all metadata. A total of 269, 16 and 110 bacterial species correlated with mean, diastolic and systolic blood pressure, respectively when we independently investigated if blood pressure parameters correlated with bacterial species (Supplementary Tables 9–11).

### 3.12 Faecal short-chain fatty acid concentrations

**Fig. 9:**
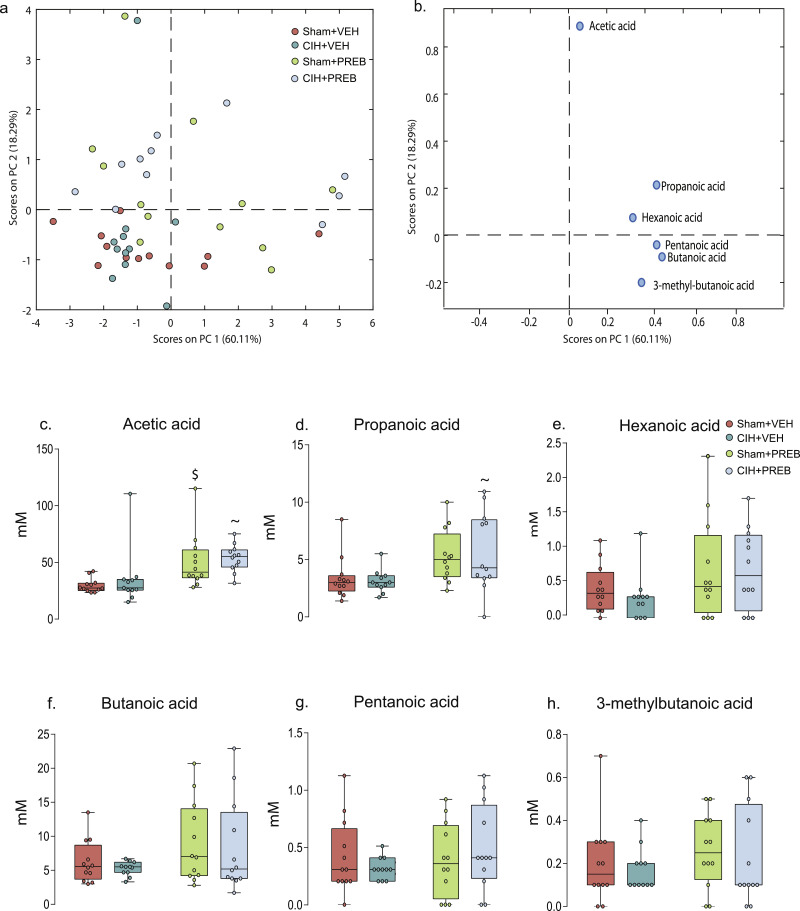
Prebiotic administration increases faecal acetic and propanoic acid Score plot (a) from principal component analysis (PCA) model calculated on the relative concentrations of detected SCFA and loading plot (b) from PCA model calculated on the relative concentrations showing which variables are responsible for the pattern observed in (a). Group data for acetic acid (c), propanoic acid (d), hexanoic acid (e), butanoic acid (f), pentanoic acid (g) and 3-methylbutanoic acid (h) in Sham+VEH, CIH+VEH, Sham+PREB and CIH+PREB. CIH, chronic intermittent hypoxia; PREB; prebiotic; VEH, vehicle. Data (c-h) are expressed as box and whisker plots (median, IQR and minimum to maximum values); *n* = 11–12. Groups (c-h) were statistically compared using two-way ANOVA, followed by Fisher's least significant difference (LSD) *post hoc* where appropriate, or non-parametric Kruskal-Wallis test, followed by Mann-Whitney U test*,* where appropriate. Acetic (*p<*0.0005; Fig. 8c) and propanoic acid (*p* = 0.011; Fig. 8d) were increased as a result of prebiotic supplementation. All other SCFAs were not different (*p*>0.05 Fig. 8e-h). ~ *p* = 0.002, CIH+VEH *versus* CIH+PREB; $ *p* = 0.001, Sham+PREB *versus* Sham+VEH.

PCA analysis did not identify distinct clustering of CIH+VEH compared with Sham+VEH rats. However, separation of vehicle from prebiotic groups was evident (Fig. 9a). Fig. 9b demonstrates this separation is due to higher concentrations of acetic, propanoic and hexanoic acid in prebiotic groups. Further analysis revealed that prebiotic supplementation significantly influenced faecal acetic (X^2^(3) = 22.420, *p<*0.0005, Fig. 9c) and propanoic (X^2^(3) = 11.211, *p* = 0.011, Fig. 9d) concentrations. Prebiotic treatment significantly increased faecal acetic acid in all statistical comparisons (CIH+PREB *versus* CIH+VEH, *p* = 0.002; Sham+PREB *versus* Sham+VEH, *p =* 0.001; Fig. 9c), propanoic acid concentrations were increased in CIH+PREB compared with Sham+PREB rats (*p* = 0.009; Fig. 9d). There was no significant difference in other SCFA concentrations (Fig. 9e-h).

## 4 Discussion

There is a growing evidence-based consensus that the gut microbiota plays a modulatory role in physiological homoeostasis. Recent studies posit that cardiorespiratory morbidity is linked to aberrant microbiota-gut-brain axis signalling [31, 33, 35, 38]. Investigations in rodents reveal that exposure to CIH disturbs the gut microbiota [14, 21, 22]. Exposure to CIH elicits cardiorespiratory dysfunction [2, 7, 8, 63], predominantly considered to be mediated via CIH-induced carotid body sensitisation [5, 6, 64], but also suggested to relate to aberrant signalling from other sites [13, 14]. When viewed together, these observations encourage a new line of enquiry. Dysregulated microbiota-gut-brain axis signalling in CIH-exposed rodent models may play a modulatory role in cardiorespiratory disturbances evident in animal models of SDB. Manipulation of the gut microbiota via antibiotic administration/faecal microbiota transfer perturbs the gut microbiota and alters cardiorespiratory control [31]. Prebiotic administration by promoting the expansion of beneficial microbes could prove effective in the prevention of CIH-induced cardiorespiratory dysfunctions.

We sought to explore the interplay between cardiorespiratory physiology and the gut microbiota in a rat model of SDB, investigating if manipulation of the gut microbiota by prebiotic fibre administration could prevent or ameliorate cardiorespiratory dysfunctions evident in a CIH animal model. The principal novel findings of this study are: 1) CIH-exposed rats have reduced relative abundance of *Lactobacilli* species; prebiotic administration shifted the bacteria microbiota composition and diversity; 2) CIH exposure did not alter known GBMs and GMMs; prebiotic administration modulated microbial functions; 3) CIH-exposed rats developed hypertension, which prebiotics failed to prevent; 4) CIH exposure had no effect on faecal SCFA concentrations; acetic and propanoic acid were increased in prebiotic groups; 5) CIH exposure increased the apnoea index during normoxia, which was unaffected by prebiotic administration; 6) Monoamine, monoamine precursor and metabolite concentrations were unaffected by exposure to CIH; prebiotic administration had modest effects on brainstem neurochemistry; 7) Cardiorespiratory responsiveness to vagal afferent nerve stimulation was unaffected by CIH exposure; prebiotic administration had modest effects on apnoea duration; 8) CIH did not affect ventilation or metabolism; prebiotic administration increased ventilatory responsiveness to hypercapnia.

Exposure to CIH elicited hypertension and a shift in autonomic balance towards sympathetic dominance, as evident by alterations in heart rate variability and spectral analysis parameters [65, 66]. Furthermore, there was an elevated propensity for central apnoea apparent in CIH-exposed rats [4, 50, 67, 68]. There is considerable evidence supporting CIH-induced sensitisation of the carotid bodies, the principal peripheral oxygen sensors, with persistent elevation in chemo-afferent traffic to the NTS of the brainstem and resultant potentiation of sympathetic nervous outflow giving rise to hypertension [9, 10, 14, 69, 70]. Carotid body ablation prevents CIH-induced hypertension and elevations in heart rate variability indicative of cardiac autonomic dysfunction [9, 10]. Nevertheless, CIH-exposed guinea-pigs, with hypoxia-insensitive carotid bodies, have altered autonomic control of heart rate associated with modification in gut microbiota composition and diversity [14]. Moreover, exposure to severe CIH elicits sympathetic over-activity and hypertension in guinea-pigs in the absence of carotid body sensitisation [13], revealing sites beyond the carotid bodies that can contribute to the manifestation of CIH-induced hypertension.

Increased apnoea index, an observation commonly observed in CIH animal models, is proposed to manifest due to disturbances in the respiratory control network [71], [72], [73], [74]. Carotid body plasticity and altered chemoreflex responsiveness is also suggested to be a driver of respiratory instability and apnoea [5, 75, 76] and may have been a driver of apnoea in our model, although the lack of change in basal breathing and ventilatory responses to hypoxia in our study suggest a central origin. Numerous studies have recently linked the development of aberrant cardiorespiratory phenotypes, particularly hypertension, to perturbed gut microbiota, aberrant function profiles of gut microbes and altered SCFA production [31, [36], [37], [38], [77], [78], [79]].

In our study, whole-metagenome shotgun sequencing revealed novel data showing that CIH+VEH hypertensive rats have modest alterations in bacterial species of the caecum, however, gut microbial functional alterations where not different compared with Sham+VEH normotensive rats. Interestingly, *Lactobacillus rhamnosus* supplementation ameliorated CIH+HSD-induced hypertension in rats [80]. The relative abundance of *L. rhamnosus* was reduced in CIH+VEH compared with Sham+VEH, however this species did not pass the 0.5% threshold. In our study, other *Lactobacilli* species were decreased in CIH+VEH compared with Sham+VEH rats suggesting that CIH-induced reductions in the relative abundance of *Lactobacilli* species may have contributed to the development of cardiovascular and autonomic dysfunction in our study, not least the development of hypertension. However, the relative abundances of *Lactobacilli* species were not different between other respective comparisons (Sham+VEH *vs* Sham+PREB; CIH+VEH *vs* CIH+PREB; Sham+PREB *vs* CIH+PREB). Furthermore, the relative abundance of other commensal bacteria species considered relevant to cardiometabolic health, such as *Akkeramansia muciniphila* were unchanged in CIH-exposed rats [81]. If gut microbiota contribute to CIH-induced hypertension, it is more likely that a complex interplay of bacterial species contribute to the development of hypertension or the maintenance of normal blood pressure *per se*. In our study, a large number of bacterial species correlated with blood pressure parameters.

To our knowledge there are no studies using whole-genome shotgun sequencing to investigate the gut microbial functions in OSA/hypertensive rodent models or animals treated with prebiotics. This area of research is in its infancy. In humans, numerous modules, essential for the host, were reduced and enriched in hypertensive compared with normotensive patients [32, 77, 78]. In our study, GBMs and GMMs were not altered in Sham+VEH compared with CIH+VEH rats and Sham+PREB compared with CIH+PREB rats, revealing that the microbial function is unaltered as a result of CIH exposure. Many bacteria species share functional characteristics, such that microbial communities with different taxonomic composition often appear to be functionally similar [82]. To this end, it appears that the functional traits of the CIH-exposed gut microbiota are not different despite changes in *Lactobacilli* species. Prebiotics had significant effects on gut microbial functions, resulting in increases and decreases in multiple modules important to the host. For example, increases in propionate synthesis in prebiotic groups determined by GBMs and GMMs, coincides with elevated faecal propionate acid in prebiotic treated rats.

Acetate-producing bacteria taxa, such as *Holdemania* were shown to be decreased in hypertensive rodents [33, 36]. Intriguingly, probiotic or prebiotic administration prevented the progression of hypertension in an OSA+HFD rat model, increasing caecal acetate and various SCFA producing bacteria that are diminished in the hypertensive rats [33]. In our study, faecal SCFA concentrations were unaffected by CIH exposure, yet, CIH-exposure caused hypertension, revealing that depletion of faecal SCFAs is not obligatory for the development of CIH-induced hypertension. Prebiotic administration increased faecal acetic and propionic acid concentrations but had no beneficial effects on cardiovascular control; hypertension and enhanced heart rate variability remained evident in CIH+PREB rats. Prebiotic administration did not ameliorate CIH-induced hypertension revealing that elevations in faecal SCFA concentrations (or at least increases in acetic acid and propionic acid) do not protect against CIH-induced hypertension. Noteworthy, other metabolites have also been linked to cardiovascular control and the development of hypertension. These include trimethylamine-N-oxide, oxidised low-density lipoprotein, nitric oxide, as well as metabolites of tryptophan, tryptophol and kynurenine [83]. Prebiotic administration altered tryptophan degradation in both sham and CIH-exposed rats as assessed using the GBM module, but did not ameliorate hypertension, suggesting that it does not play a significant role in the development of cardiovascular morbidity in our animal model.

Our modest CIH paradigm did not increase plasma or brainstem pro-inflammatory cytokines, consistent with other studies showing no difference in systemic pro-inflammatory cytokine concentrations following exposure to CIH [80, 84]. However, others have displayed neuro- and systemic inflammation as a result of severe CIH exposure [20, [84], [85], [86]]. Differences are likely due to pattern, duration and intensity of CIH exposure. In rodents, exposure to CIH has been shown to increase plasma lipopolysaccharide (LPS) and elevate gut inflammation, contributing to intestinal barrier dysfunction [21, 87], a phenotype also evident in other hypertensive models [32, 79]. Although, we did not examine intestinal function in our study, given that plasma inflammatory mediators were not affected by CIH in our model, we suggest that gut barrier integrity was maintained. Hence, we suggest that systemic inflammation did not contribute to the development of CIH-induced cardiorespiratory dysfunction in our study. This does not preclude possible involvement of microbiota-gut-brain axis signalling in the elaboration of cardiorespiratory dysfunction in other models and in OSA. Notably, faecal microbiota transfer from donor normotensive rats to hypertensive recipient rats, and prebiotic and probiotic administration each independently decrease blood pressure, and prevent intestinal dysfunction and neuroinflammation in hypertensive models, including animal models of OSA [33, 38, 80].

Prebiotic administration increased chemoreflex control of breathing in response to hypercapnia and hypoxic hypercapnia. Hypercapnia is primarily sensed by central chemoreceptors residing in the brainstem. Increased ventilatory responsiveness to hypercapnia is particularly interesting given that rats exposed to pre-natal stress exhibit altered ventilatory responsiveness to hypoxic and hypercapnic chemostimulation in adulthood, which correlated with changes in the gut microbiota [29]. Moreover, antibiotic administration and faecal microbiota transfer were shown to perturb the gut microbiota composition and blunt chemoreflex control of breathing [31]. The latter observation combined with findings from the present study suggests that the gut microbiota may shape brainstem responsiveness to carbon dioxide (acidosis) with implications for a range of respiratory control disorders.

We assessed monoamines and monoamine metabolites and precursors in the pons and medulla oblongata of the brainstem that are crucial in the neuromodulation of cardiorespiratory control. No significant modifications in monoamine, metabolite and precursor concentrations were evident in the brainstem of CIH-exposed rats. There was a trend for reduced dopamine turnover in the medulla oblongata of Sham+PREB rats, with elevated ventilatory responses to hypercapnia and hypoxic hypercapnia compared with Sham+VEH rats. This finding is particularly interesting given that rats that received antibiotics or faecal microbiota transfer, each resulting in perturbed gut microbiota, display blunted ventilatory responses to hypercapnia and exhibit increased brainstem dopamine turnover [31]. Intravenous administration of a D1 receptor agonist increases reactivity to carbon dioxide [88], therefore perhaps D1 receptor activation underpins elevated ventilatory responses to chemostimulation in Sham+PREB rats. Previous studies demonstrate that prebiotic administration affected DOPAC concentrations in the brainstem and frontal cortex of mice [89]. Yet, prebiotic administration did not alter other monoamine, metabolites and precursors of the dopaminergic pathway in animal models [89, 90]. CIH+PREB rats revealed significantly increased 5-HIAA concentrations in the medulla oblongata compared with CIH+VEH rats. Noteworthy, lesions of raphé serotonergic neurons and transgenic rodents without 5-HT neurones display reduced respiratory responsiveness to hypercapnic chemostimulation [91], [92], [93], [94]. However, serotonin turnover was not different in CIH+PREB rats compared with CIH+VEH. It is not likely that serotonin mediated the elevated ventilatory drive to breathe in response to hypercapnia associated with prebiotic supplementation. Altered 5-HT receptor levels and 5-HT concentrations have been observed in the rodent pre-frontal cortex after prebiotic administration [89, 95]. It is unlikely that 5-HT is implicated in the ventilatory changes described in this study. However, decreased dopamine turnover in Sham+PREB rats may be related to increased ventilatory responses to hypercapnia and hypoxic hypercapnia.

The afferent vagal pathway is a pivotal signalling pathway of the microbiota-gut-brain axis, which responds to various stimuli including cytokines, bacterial metabolites including SCFAs, gut hormones and neurotransmitters [97], [97], [98], [99], [100]. Central integration of vagal afferent signals occurs within the NTS of the brainstem. PBG, which is a 5-HT_3_ receptor agonist, activates pulmonary vagal afferent C-fibres manifesting the pulmonary chemoreflex characterised by decreased blood pressure, bradycardia, apnoea and post apnoea-induced tachypnoea. Exposure to CIH did not affect the pulmonary chemoreflex, but prebiotic administration increased apnoea duration. *Post hoc* analysis determined that there were no statistically significant differences between groups suggesting that vagal influence over these critical control centres was unaltered by any potential changes in microbiota-gut-brain signalling. Of interest, cardiorespiratory responses to PBG were also unaffected in other models of disrupted gut microbiota [31]. Thus, notwithstanding alterations to the gut microbiota in CIH+VEH rats, as well as the notable changes in the gut microbiota, SCFA concentrations, microbial functional characteristics and brainstem neurochemistry in prebiotic groups, no major differences in cardiorespiratory efferent responses to vagal afferent stimulation were observed revealing intact pulmonary chemoreflex circuit function in CIH-exposed and prebiotic supplemented animals. This does not preclude however, the possibility of altered vagal signalling from the gut in our models, which warrants attention in future studies.

## 5 Limitations

By design, we utilised a modest CIH paradigm sufficient to induce hypertension and apnoea. Our rationale was to utilise the minimum “dose” of CIH to evoke the classic aberrant cardiorespiratory phenotypes and consider the effects of this paradigm on the gut microbiota. Moreover we explored the potential capacity for prebiotic administration, sufficient to modulate gut microbial function, to ameliorate CIH-induced cardiorespiratory outcomes in this model. Our focus was to consider the extent to which changes in the gut microbiota were obligatory for OSA-related cardiorespiratory impairment, principally focussed on CIH-induced hypertension. Notwithstanding our findings, revealing no obligatory role for microbiota-gut-brain axis signalling in the development of cardiorespiratory morbidity in our model, the results do not preclude a potential role for the microbiota-gut-brain axis in OSA-related cardiorespiratory morbidity. A more severe CIH paradigm with variations in the frequency of cycles, intensity of hypoxia and/or duration of cumulative exposure would likely elicit robust effects on the microbiota, with altered functional characteristics. In this way, it is plausible to suggest that the microbiota-gut-brain axis could be implicated in the elaboration of cardiorespiratory morbidity, and therefore could yet be a potential therapeutic target. Our data point to mechanisms independent of the gut-brain axis in the development of CIH-related morbidities, most likely related to carotid body sensitisation, but they do not exclude the possibility of an important contribution by the gut-brain axis in the maintenance and progression of cardiorespiratory morbidity in circumstances wherein CIH evokes disruption to the gut microbiota and gut barrier permeability.

We examined only one prebiotic combination at one dose for one period in our study. It would be of interest to investigate the effects of different prebiotic strategies on cardiorespiratory control in CIH models. Additionally, other microbiota interventions such as probiotics or faecal microbiota transfer may prove efficacious in preventing CIH-induced hypertension. Indeed, *L. rhamnosus* and normotensive faecal matter have been shown to be beneficial in animal models of OSA-induced hypertension [38, 80]. However, it is worth recognising that precautions should be taken when using faecal microbiota transfer and probiotic strains. For example, faecal microbiota transfer has been shown to blunt ventilatory responses to elevated carbon dioxide in rats [31]. *Lactobacilli* probiotic species have been associated with adverse outcomes in humans including abscesses, pneumonia and sepsis [101]. Further work is required to investigate if different microbiota intervention strategies have beneficial effects on cardiorespiratory control in CIH-exposed rats.

Whereas we assessed inflammatory cytokines in plasma as an index of global inflammation, we did not examine intestinal permeability, which is altered in severe models of CIH exposure [22, 87]. Differences in our findings compared with others may relate to differences in intestinal barrier function and integrity. As systemic inflammation was not evident in CIH-exposed rats in our study, we posit that intestinal permeability is not likely altered in our animal model, but this remains to be determined.

We measured faecal SCFA concentrations but these do not necessarily represent concentrations in plasma and target tissues, such as the gut or brain. The functions of SCFAs depend upon activation of transmembrane G-protein coupled receptors [102]. We did not explore SCFA receptor expression or downstream signalling in target tissues of interest. We acknowledge that this is a limitation of our study. In particular, regarding prebiotic strategies, our study does not rule out the possibility that strategies that are effective in eliciting SCFA-dependant signalling in one or more sites could yet prove beneficial in protecting against CIH-induced cardiorespiratory dysfunction in our model, as has been shown in other models.

## 6 Conclusion

Our novel findings add to the growing understanding of the role of the microbiota-gut-brain axis in the control of breathing and cardiovascular function [31, 33, 80]. Herein we confirm that CIH exposure leads to the development of adverse cardiorespiratory and autonomic control, resulting in hypertension, cardiac autonomic imbalance and elevated propensity for apnoea. We revealed for the first time using whole-metagenome shotgun sequencing that *Lactobacilli* species were decreased in CIH-exposed rats, but gut microbial functional characteristics were unaltered. Faecal SCFA concentrations were not altered by CIH exposure. Prebiotics increased faecal SCFAs and modulated GBMs and GMMs but were not effective in preventing CIH-induced cardiorespiratory dysfunction. Interestingly, ventilatory responses to hypercapnic and hypoxic hypercapnic chemostimulation were altered in prebiotic treated groups. Significant modulations to the gut microbiota may shape brainstem responsiveness to acidosis which has implications for homoeostatic function of integrative body systems. Our findings extend previous knowledge of the relationship between the gut microbiota and cardiorespiratory control in OSA animal models. Our results demonstrate that in a relatively mild model of CIH, sufficient to evoke classic cardiorespiratory dysfunction, changes in gut microbial function are not obligatory for the development of morbidity. However, our study does not exclude the potential for microbiota-gut-brain axis involvement in severe CIH models and hence OSA, wherein the microbiota-gut-brain axis may be relevant in the elaboration and maintenance of cardiorespiratory morbidity with progressive disease.

## Competing interests

JFC is in receipt of research funding from 4D-Pharma, Mead Johnson, Nutricia, Dupont and Cremo and has been an invited speaker at meetings organised by Mead Johnson, Alkermes, Abbott Nutrition, Danone Nutricia and Janssen. GC is in receipt of research funding from Pharmavite and has been an invited speaker at meetings organised by Janssen and Probi. All other authors report no financial, professional or personal conflicts of interest relating to this publication.

## Authors’ contributions

KMO'C; experimental design; acquisition of data; data and statistical analysis and interpretation of data; drafting of the original manuscript; EFL: *in vivo* studies: experimental design; acquisition of data; interpretation of physiological data; drafting of the original manuscript; TB: whole-metagenome shotgun sequencing: functional annotations; microbiota data and statistical analysis; interpretation of microbial data; EFL and TB contributed equally to the study; VLP: metagenomic bioinformatic analysis; FC: DNA extraction and whole-metagenome shotgun sequencing; PC: whole-metagenome shotgun sequencing: critical revision of the manuscript for important intellectual content; GC: HPLC studies: interpretation of monoamine data; critical revision of the manuscript for important intellectual content; JFC: critical revision of the manuscript for important intellectual content; KDO'H: experimental design; interpretation of physiological data; drafting and critical revision of the manuscript for important intellectual content.

## Funding

This project was funded by the Department of Physiology, and the APC Microbiome Ireland (funded by Science Foundation Ireland (SFI/ 12/RC/2273 P2), University College Cork, Ireland. The institution had no role in the study design, data collection, data analysis, interpretation or writing of the manuscript.

## Appendix Supplementary materials

Image, application 1

Image, application 2

## Footnotes

Supplementary material associated with this article can be found in the online version at doi:10.1016/j.ebiom.2020.102968.
