# Supplementary material for: Prebiotic administration modulates gut microbiota and faecal short-chain fatty acid concentrations but does not prevent chronic intermittent hypoxia-induced apnoea and hypertension in adult rats
Source: eBioMedicine. 2020 Aug 30;59:102968. doi: 10.1016/j.ebiom.2020.102968 (PMC7475129; doi:10.1016/j.ebiom.2020.102968)
Supplement: Supplementary file 2 [file mmc2.docx]

***Supplementary table 1. Body and tissue weights***

|  | Sham+VEH | CIH+VEH | Sham+PREB | CIH+PREB | p-value  (Kruskal-Wallis) | p-value (two-way ANOVA) | Sham+VEH v CIH+VEH | CIH+VEH v CIH+PREB | Sham+PREB v CIH+PREB | Sham+VEH v Sham+PREB |
| --- | --- | --- | --- | --- | --- | --- | --- | --- | --- | --- |
| RV (mg) | 169 ± 31 | 174 ± 20 | 179 ± 32 | 161 ± 26 | N/A | Diet, p=0.627; Exposure, p=0.344; Diet*Exposure, p=0.121 | - | - | - | - |
| LV (mg) | 864 ± 73 | 799 ± 41 | 730 ± 41 | 621 ± 72 | N/A | Diet, **p<0.0005**; Exposure, **p<0.0005**; Diet*Exposure, **p=0.023** | 0.207 | **<0.0005** | **<0.0005** | **<0.0005** |
| LV+RV (mg) | 1025 ± 60 | 997 ± 38 | 917 ± 61 | 781 ± 94 | **<0.0005** | N/A | 0.074 | **<0.0005** | **0.001** | **0.001** |
| Caecum (g) | 3.6 ± 1.0 | 3.0 ± 0.3 | 6.4 ± 1.2 | 6.0 ± 1.8 | N/A | Diet, **p<0.0005**; Exposure, p=0.428; Diet*Exposure, p=0.926 | 0.565 | **<0.0005** | 0.583 | **<0.0005** |
| Lung dry weight (mg) | 316 ± 38 | 340 ± 13 | 296 ± 21 | 305 ± 52 | **0.001** | N/A | 0.052 | **0.012** | 0.862 | **0.009** |
| Lung wet weight (mg) | 1422 ± 146 | 1596 ± 182 | 1394 ± 112 | 1390 ± 244 | **0.015** | N/A | 0.206 | 0.024 | 0.908 | 0.035 |

RV, right ventricle; LV, left ventricle; CIH, chronic intermittent hypoxia; PREB, prebiotic; VEH, vehicle. Data are shown as mean ± SD and were statistically compared using two-way ANOVA, followed by Fisher’s least significant difference (LSD) *post hoc* where appropriate, or non-parametric Kruskal-Wallis test, followed by Mann-Whitney U test*,* where appropriate. Statistical significance for multiple comparisons was accepted at p<0.05 divided by the number of comparisons made, which was four i.e. p<0.0125. *p*-values shown in **bold** highlight significant differences.

***Supplementary table 2. Baseline ventilation in behaving rats during quiet rest***

|  | Sham+VEH | CIH+VEH | Sham+PREB | CIH+PREB | p-value  (Kruskal-Wallis) | p-value (two-way ANOVA) | Sham+VEH v CIH+VEH | CIH+VEH v CIH+PREB | Sham+PREB v CIH+PREB | Sham+VEH v Sham+PREB |
| --- | --- | --- | --- | --- | --- | --- | --- | --- | --- | --- |
| V_E_ (ml/min) | 28 ± 3 | 29 ± 4 | 31 ± 5 | 34 ± 5 | N/A | Diet, **p=0.004**; Exposure, p=0.107; Diet*Exposure, p=0.605 | 0.436 | 0.015 | 0.129 | 0.074 |
| V_T_ (ml) | 2.5 ± 0.3 | 2.5 ± 0.3 | 2.4 ± 0.4 | 2.3 ± 0.3 | 0.667 | N/A | - | - | - | - |
| V_T_/T_i_ (ml/s) | 10 ± 1.9 | 9.2 ± 1.2 | 9.5 ± 1.5 | 8.7 ± 1.0 | N/A | Diet, p=0.076; Exposure, **p=0.024**; Diet*Exposure, p=0.636 | 0.055 | 0.353 | 0.188 | 0.108 |
| VO_2_ (ml/min) | 9.8 ± 2.4 | 8.5 ± 2.8 | 8.6 ± 2.8 | 8.6 ± 2.8 | N/A | Diet, p=0.472; Exposure, p=0.385; Diet*Exposure, p=0.385 | - | - | - | - |
| VCO_2_ (ml/min) | 7.0 ± 0.9 | 6.6 ± 0.8 | 5.7 ± 1.0 | 5.5 ± 0.8 | **0.001** | N/A | 0.218 | **0.004** | 0.795 | **0.006** |

V_E_, minute ventilation; *V*_T,_ tidal volume; *V*_T_/T_i_, mean inspiratory flow; *V*O_2_, oxygen consumption; *V*CO_2,_ carbon dioxide production; V_E_/ CIH, chronic intermittent hypoxia; PREB, prebiotic; VEH, vehicle. Data are shown as mean ± SD and were statistically compared using two-way ANOVA, followed by Fisher’s least significant difference (LSD) *post hoc* where appropriate, or non-parametric Kruskal-Wallis test, followed by Mann-Whitney U test*,* where appropriate. Statistical significance for multiple comparisons was accepted at p<0.05 divided by the number of comparisons made, which was four i.e. p<0.0125. *p*-values shown in **bold** highlight significant differences.

***Supplementary table 3. Cardiorespiratory responsiveness to chemostimulation in urethane anaesthetised rats***

|  | Sham+VEH | CIH+VEH | Sham+PREB | CIH+PREB | p-value  (Kruskal-Wallis) | p-value (two-way ANOVA) | Sham+VEH v CIH+VEH | CIH+VEH v CIH+PREB | Sham+PREB v CIH+PREB | | Sham+VEH v Sham+PREB |
| --- | --- | --- | --- | --- | --- | --- | --- | --- | --- | --- | --- |
| *Hypoxia (% change from baseline)* | | | | | |  |  |  |  |  | |
| f_R_ | 20 ± 13 | 23 ± 9 | 12 ± 17 | 16 ± 23 | N/A | Diet, p=0.120; Exposure, p=0.697; Diet*Exposure, p=0.774 | - | - | - | | - |
| V_T_ | 8 ± 14 | 15 ± 7 | 14 ± 10 | 10 ± 14 | N/A | Diet, p=0.976; Exposure, p=0.687; Diet*Exposure, p=0.137 | - | - | - | | - |
| V_E_ | 30 ± 28 | 42 ± 15 | 28 ± 25 | 29 ± 38 | 0.815 | N/A | - | - | - | | - |
| MAP | -37 ± 12 | -31 ± 15 | -38 ± 11 | -31 ± 15 | N/A | Diet, p=0.685; Exposure, p=0.398; Diet*Exposure, p=0.431 | - | - | - | | - |
| DBP | -44 ± 15 | -46 ± 10 | -45 ± 13 | -40 ± 18 | N/A | Diet, p=0.842; Exposure, p=0.682; Diet*Exposure, p=0.424 | - | - | - | | - |
| SBP | -25 ± 10 | -18 ± 10 | -20 ± 9.8 | -14 ± 11 | 0.177 | N/A | - | - | - | | - |
| HR | 2 ± 10 | 4 ± 8 | 4 ± 6 | 4 ± 13 | N/A | Diet, p=0.633; Exposure, p=0.494; Diet*Exposure, p=0.990 | - | - | - | | - |
| Hypoxic hypercapnia (% change from baseline) | | | | |  |  |  |  |  |  | |
| f_R_ | 38 ± 17 | 33 ± 13 | 25 ± 24 | 17 ± 23 | **0.023** | N/A | 0.944 | **0.012** | 0.262 | | 0.205 |
| V_T_ | 67 ± 17 | 63 ± 17 | 68 ± 28 | 84 ± 23 | 0.056 | N/A | - | - | - | | - |
| V_E_ | 128 ± 30 | 114 ± 23 | 112 ± 60 | 115 ± 56 | N/A | Diet, p=0.644; Exposure, p=0.741; Diet*Exposure, p=0.611 | - | - | - | | - |
| MAP | -33 ± 12 | -22 ± 15 | -21 ± 15 | -9 ± 13 | N/A | Diet, **p=0.010**; Exposure, **p=0.008**; Diet*Exposure, p=0.693 | 0.116 | 0.036 | 0.033 | | 0.211 |
| DBP | -40 ± 15 | -29 ± 18 | -29 ± 20 | -13 ± 18 | N/A | Diet, **p=0.023**; Exposure, **p=0.011**; Diet*Exposure, p=0.667 | 0.100 | 0.052 | 0.028 | | 0.183 |
| SBP | -10 ± 7 | -7 ± 12 | -6 ± 10 | -2 ± 5 | 0.173 | N/A | - | - | - | | - |
| HR | 6 ± 4 | 8 ± 4 | 9 ± 6 | 8 ± 8 | N/A | Diet, p=0.619; Exposure, p=0.889; Diet*Exposure, p=0.590 | - | - | - | | - |
| Sodium cyanide (% change from baseline) | | | | |  |  |  |  |  |  | |
| f_R_ | 53 ± 18 | 51 ± 20 | 62 ± 21 | 44 ± 24 | 0.213 | N/A | - | - | - | | - |
| V_T_ | 46 ± 12 | 45 ± 12 | 51 ± 15 | 44 ± 14 | N/A | Diet, p=0.557; Exposure, p=0.311; Diet*Exposure, p=0.474 | - | - | - | | - |
| V_E_ | 110 ± 34 | 112 ± 38 | 133 ± 41 | 102 ± 49 | 0.489 | N/A | - | - | - | | - |
| MAP | 19 ± 8 | 18 ± 8 | 19 ± 8 | 17 ± 13 | N/A | Diet, p=0.383; Exposure, p=0.728; Diet*Exposure, p=0.652 | - | - | - | | - |
| DBP | 22 ± 9 | 26 ± 23 | 28 ±17 | 28 ± 25 | N/A | Diet, p=0.489; Exposure, p=0.596; Diet*Exposure, p=0.823 | - | - | - | | - |
| SBP | 12 ± 8 | 8 ± 7 | 8 ± 8 | 8 ± 6 | 0.131 | N/A | - | - | - | | - |
| HR | 7 ± 5 | 7 ± 6 | 8 ± 7 | 9 ± 9 | N/A | Diet, p=0.960; Exposure, p=0.732; Diet*Exposure, p=0.994 | - | - | - | | - |

*f_R_,* respiratory frequency (brpm, breaths per min); *V_T_*_,_ tidal volume; *V_E_,* minute ventilation; MAP, mean arterial pressure; DBP, diastolic blood pressure; SBP, systolic blood pressure; HR, heart rate (bpm, beats per min); CIH, chronic intermittent hypoxia; preb, prebiotic; VEH, vehicle. Data are shown as mean ± SD and were statistically compared using two-way ANOVA, followed by Fisher’s least significant difference (LSD) *post hoc* where appropriate, or non-parametric Kruskal-Wallis test, followed by Mann-Whitney U test*,* where appropriate. Statistical significance for multiple comparisons was accepted at p<0.05 divided by the number of comparisons made, which was four i.e. p<0.0125. *p*-values shown in **bold** highlight significant differences.

***Supplementary table 4. Baroreflex receptor sensitivity in urethane anaesthetised rats***

|  | Sham+VEH | CIH+VEH | Sham+PREB | CIH+PREB | p-value  (Kruskal-Wallis) | p-value (two-way ANOVA) | Sham+VEH v CIH+VEH | CIH+VEH v CIH+PREB | Sham+PREB v CIH+PREB | | Sham+VEH v Sham+PREB |
| --- | --- | --- | --- | --- | --- | --- | --- | --- | --- | --- | --- |
| Phenylephrine (% change from baseline) | | | | |  |  |  |  |  | |  |
| SBP | 43 ± 11 | 47 ± 16 | 35 ± 12 | 37 ± 9 | **0.018** | N/A | 0.999 | 0.015 | 0.667 | | 0.049 |
| HR | -25 ± 14 | -24 ± 14 | -20 ± 12 | -15 ± 7 | 0.149 | N/A | - | - | - | | - |
| Sodium nitroprusside (% change from baseline) | | | | | |  |  |  |  |  |  |
| SBP | -30 ± 14 | -31 ± 14 | -22 ± 10 | -27 ± 11 | 0.236 | N/A | - | - | - | | - |
| HR | 13 ± 5 | 8 ± 3 | 6 ± 4 | 7 ± 3 | N/A | Diet, **p=0.001**; Exposure, p=0.106; Diet*Exposure, p=0.053 | 0.013 | 0.245 | 0.842 | | **<0.0005** |

SBP, systolic blood pressure; HR, heart rate; CIH, chronic intermittent hypoxia; PREB, prebiotic; VEH, vehicle. Data are shown as mean ± SD and were statistically compared using two-way ANOVA, followed by Fisher’s least significant difference (LSD) *post hoc* where appropriate, or non-parametric Kruskal-Wallis test, followed by Mann-Whitney U test*,* where appropriate. Statistical significance for multiple comparisons was accepted at p<0.05 divided by the number of comparisons made, which was four i.e. p<0.0125. *p*-values shown in **bold** highlight significant differences.

***Supplementary table 5. Diaphragm EMG activity during baseline and chemostimulation in urethane anaesthetised rats***

|  | Sham+VEH | CIH+VEH | Sham+PREB | CIH+PREB | p-value  (Kruskal-Wallis) | p-value (two-way ANOVA) | Sham+VEH v CIH+VEH | CIH+VEH v CIH+PREB | Sham+PREB v CIH+PREB | | Sham+VEH v Sham+PREB |
| --- | --- | --- | --- | --- | --- | --- | --- | --- | --- | --- | --- |
| Baseline |  |  |  |  |  |  |  |  |  | |  |
| Area under the curve (v.s) | 0.02 ± 0.00 | 0.03 ± 0.01 | 0.03 ± 0.02 | 0.03 ± 0.03 | 0.222 | N/A | - | - | - | | - |
| Max slope (v/s) | 1.0 ± 0.2 | 1.1 ± 0.3 | 1.4 ± 0.5 | 1.5 ± 0.7 | N/A | Diet, **p=0.023**; Exposure, p=0.482; Diet*Exposure, p=0.846 | 0.715 | 0.074 | 0.531 | | 0.138 |
| Occlusion (% change from baseline) | | | | | |  |  |  |  |  | |
| Area under the curve | 87 ± 40 | 65 ± 34 | 96 ± 44 | 57 ± 41 | 0.073 | N/A | - | - | - | | - |
| Hypoxia (% change from baseline) | | | |  |  |  |  |  |  |  | |
| Area under the curve | 11 ± 21 | 6 ± 13 | 12 ± 11 | 9 ± 12 | 0.974 | N/A | - | - | - | | - |
| Max slope | 31 ± 28 | 19 ± 14 | 22 ± 17 | 24 ± 19 | 0.511 | N/A | - | - | - | | - |
| Hypoxic hypercapnia (% change from baseline) | | | | | | |  |  |  |  | |
| Area under the curve | 18 ± 15 | 11 ± 16 | 18 ± 15 | 26 ± 13 | N/A | Diet, p=0.085; Exposure, p=0.967; Diet*Exposure, p=0.159 | - | - | - | | - |
| Max slope | 47 ± 17 | 39 ± 21 | 32 ± 23 | 59 ± 37 | N/A | Diet, p=0.216; Exposure, p=0.085; Diet*Exposure, p=0.829 | - | - | - | | - |
| Sodium cyanide (% change from baseline) | | | | | |  |  |  |  |  | |
| Area under the curve | 23 ± 12 | 30 ± 11 | 46 ± 25 | 47 ± 30 | N/A | Diet, **p=0.003**; Exposure, p=0.652; Diet*Exposure, p=0.639 | 0.511 | 0.063 | 0.989 | | 0.015 |
| Max slope | 70 ± 35 | 66 ± 17 | 67 ± 23 | 64 ± 33 | N/A | Diet, p=0.829; Exposure, p=0.669; Diet*Exposure, p=0.922 | - | - | - | | - |

CIH, chronic intermittent hypoxia; PREB, prebiotic; VEH, vehicle. Data are shown as mean ± SD and were statistically compared using two-way ANOVA, followed by Fisher’s least significant difference (LSD) *post hoc* where appropriate, or non-parametric Kruskal-Wallis test, followed by Mann-Whitney U test*,* where appropriate. Statistical significance for multiple comparisons was accepted at p<0.05 divided by the number of comparisons made, which was four i.e. p<0.0125. *p*-values shown in **bold** highlight significant differences.

***Supplementary table 6. Blood gases in urethane anaesthetised rats***

|  | Sham+VEH | CIH+VEH | Sham+PREB | CIH+PREB | p-value  (Kruskal-Wallis) | p-value (two-way ANOVA) | Sham+VEH v CIH+VEH | CIH+VEH v CIH+PREB | Sham+PREB v CIH+PREB | Sham+VEH v Sham+PREB |
| --- | --- | --- | --- | --- | --- | --- | --- | --- | --- | --- |
| pH | 7.37 ± 0.04 | 7.35 ± 0.04 | 7.40 ± 0.02 | 7.36 ± 0.04 | N/A | Diet, p=0.436; Exposure, p=0.152; Diet*Exposure, p=0.301 | - | - | - | - |
| PaCO_2_ (mmHg) | 41 ± 13 | 48 ± 5 | 50 ± 4 | 48 ± 6 | 0.140 | N/A | - | - | - | - |
| PaO_2_ (mmHg) | 95 ± 6 | 98 ± 7 | 100 ± 11 | 106 ± 10 | N/A | Diet, **p=0.033**; Exposure, p=0.100; Diet*Exposure, p=0.689 | 0.370 | 0.817 | 0.149 | **0.002** |
| Haematocrit (%) | 47.5 ± 2.5 | 46.5 ± 3.0 | 43.1 ± 2.8 | 43.9 ± 4.0 | N/A | Diet, **p<0.0005**; Exposure, p=0.871; Diet*Exposure, p=0.305 | 0.394 | 0.050 | 0.544 | **0.001** |
| [Hb] (g/dl) | 16.2 ± 0.9 | 15.2 ± 1.0 | 14.6 ± 0.9 | 14.9 ± 1.3 | N/A | Diet, **p<0.0005**; Exposure, p=0.943; Diet*Exposure, p=0.324 | 0.447 | 0.047 | 0.521 | **0.002** |

Pco_2_, partial pressure of arterial carbon dioxide_;_ Pao_2_, partial pressure of arterial oxygen; [Hb], haemoglobin concentration; MAP, mean arterial blood pressure; SBP, systolic blood pressure; DBP, diastolic blood pressure; HR, heart rate (bpm, beat per min); CIH, chronic intermittent hypoxia; PREB, prebiotic; VEH, vehicle. Data are shown as mean ± SD and were statistically compared using two-way ANOVA, followed by Fisher’s least significant difference (LSD) *post hoc* where appropriate, or non-parametric Kruskal-Wallis test, followed by Mann-Whitney U test*,* where appropriate. Statistical significance for multiple comparisons was accepted at p<0.05 divided by the number of comparisons made, which was four i.e. p<0.0125. *p*-values shown in **bold** highlight significant differences.

***Supplementary table 7. Body and tissue weights in rats euthanized by pentobarbitone***

|  | Sham+VEH | CIH+VEH | p-value |
| --- | --- | --- | --- |
| Body mass  (g) | 353 ±19 | 343 ±22 | 0.287 |
| RV (mg) | 215 ± 17 | 182 ± 32 | 0.506 |
| RV  (mg/100g) | 61 ± 6 | 56 ± 8 | 0.419 |
| LV (mg) | 803 ± 51 | 787 ± 71 | 0.844 |
| LV  (mg/100g) | 228 ± 16 | 244 ± 21 | 0.311 |
| RV+LV (mg) | 1018 ± 42 | 2112 ± 100 | 0.634 |
| RV+LV (mg/100g) | 290 ± 17 | 300 ± 27 | 0.172 |
| Caecum (g) | 5.2 ± 1.0 | 5.0 ± 1.6 | 0.710 |
| Caecum (g/100g) | 1.5 ± 0.3 | 1.5 ± 0.5 | 0.496 |

BW, body weight; RV, right ventricle; LV, left ventricle; CIH, chronic intermittent hypoxia; VEH, vehicle. Data are shown as mean ± SD and were statistically compared using independent samples *t*-test or non-parametric Mann-Whitney U test*,* where appropriate.

***Supplementary table 8. Cytokine concentrations in pons and medulla oblongata***

|  | Sham+VEH | CIH+VEH | p-value |
| --- | --- | --- | --- |
| Pons |  |  |  |
| IFN-γ | 7.7 ± 0.6 | 7.8 ± 0.7 | 0.097 |
| IL-1β | 253 ± 26 | 275 ± 60 | 0.518 |
| IL-4 | 2.2 ± 0.0008 | 2.2 ± 0.0008 | 0.131 |
| IL-5 | 21 ± 0.8 | 20 ± 0.6 | **0.044** |
| IL-6 | 38 ± 11.0 | 36 ± 7.1 | 0.756 |
| KC/GRO | 4.1 ± 0.9 | 4.2 ± 1.2 | 0.621 |
| IL-10 | 4.3 ± 1.5 | 4.1 ± 1.8 | 0.902 |
| IL-13 | 1.8 ± 0.04 | 1.8 ± 0.05 | 0.163 |
| TNF-α | 0.6 ± 0.04 | 0.6 ± 0.03 | 0.843 |
| Medulla |  |  |  |
| IFN-γ | 8.1 ± 0.9 | 7.6 ± 1.3 | 0.050 |
| IL-1β | 283 ± 27 | 268 ± 33 | 0.057 |
| IL-4 | 2.2 ± 0.0006 | 2.2 ± 0.0006 | 0.070 |
| IL-5 | 21 ± 0.5 | 20 ± 0.4 | **0.016** |
| IL-6 | 48 ± 18 | 33 ± 10 | 0.380 |
| KC/GRO | 4.7 ± 1.2 | 3.6 ± 1.4 | 0.052 |
| IL-10 | 4.8 ± 1.8 | 3.0 ± 1.5 | **0.035** |
| IL-13 | 1.8 ± 0.08 | 1.8 ± 0.06 | 0.906 |
| TNF-α | 0.6 ± 0.05 | 0.6 ± 0.04 | 0.373 |

IFN-y, interferon-y; IL-β, interleukin-β; IL-4, [interleukin-4](https://www.sciencedirect.com/topics/medicine-and-dentistry/interleukin-4); IL-5, interleukin-5; IL-6, interleukin-6; KC/GRO, [keratinocyte](https://www.sciencedirect.com/topics/medicine-and-dentistry/keratinocyte) chemoattractant/growth-related [oncogene](https://www.sciencedirect.com/topics/medicine-and-dentistry/oncogene); IL-10, [interleukin-10](https://www.sciencedirect.com/topics/medicine-and-dentistry/interleukin-10); [IL-13](https://www.sciencedirect.com/topics/medicine-and-dentistry/interleukin-13), [interleukin-13](https://www.sciencedirect.com/topics/medicine-and-dentistry/interleukin-13); [TNF-α](https://www.sciencedirect.com/topics/medicine-and-dentistry/tumor-necrosis-factor), [tumour necrosis factor](https://www.sciencedirect.com/topics/medicine-and-dentistry/tumor-necrosis-factor); CIH, chronic intermittent hypoxia; VEH, vehicle. Data are shown as mean ± SD and were statistically compared using independent samples *t*-test or non-parametric Mann-Whitney U test*,* where appropriate. *p*-values shown in **bold** highlight significant differences.

***Supplementary table 9. Significant correlations between mean arterial blood pressure and bacterial species.***

| Bacterial species | p-value | Statistic | q-value |
| --- | --- | --- | --- |
| Actinobacteria |  |  |  |
| Acidimicrobium ferrooxidans | 0.000104 | -0.55375 | 0.01888 |
| Acidipropionibacterium sp. JS278 | 0.001673 | -0.45942 | 0.04825 |
| Actinomyces oris | 0.005244 | -0.41199 | 0.0711 |
| Actinomyces sp. VUL4_3 | 0.00688 | -0.39974 | 0.078342 |
| Arsenicicoccus sp. oral taxon 190 | 0.007161 | -0.39789 | 0.080043 |
| Arthrobacter sp. ATCC 21022 | 0.005547 | -0.40949 | 0.072506 |
| Arthrobacter sp. PGP41 | 0.011805 | -0.37404 | 0.090492 |
| Arthrobacter sp. U41 | 0.000944 | -0.48103 | 0.038102 |
| Bifidobacterium angulatum | 0.0069 | -0.3996 | 0.078342 |
| Bifidobacterium asteroides | 0.003057 | -0.43518 | 0.05565 |
| Candidatus Rhodoluna planktonica | 0.000625 | 0.495784 | 0.036179 |
| Cellulomonas fimi | 0.010572 | -0.37945 | 0.089473 |
| Cellulomonas sp. PSBB021 | 0.005167 | -0.41265 | 0.0711 |
| Coriobacterium glomerans | 0.001526 | -0.46298 | 0.047807 |
| Corynebacterium callunae | 0.008412 | -0.39038 | 0.084904 |
| Corynebacterium cystitidis | 0.000753 | -0.4892 | 0.037892 |
| Corynebacterium flavescens | 0.007732 | -0.39433 | 0.082378 |
| Corynebacterium imitans | 0.012954 | -0.36943 | 0.094512 |
| Corynebacterium jeikeium | 0.001214 | -0.47167 | 0.041579 |
| Corynebacterium provencense | 0.010233 | -0.38103 | 0.089069 |
| Corynebacterium sp. ATCC 6931 | 0.002961 | -0.4365 | 0.05565 |
| Corynebacterium ulcerans | 0.002466 | -0.44401 | 0.052096 |
| Dermacoccus nishinomiyaensis | 0.008845 | -0.38801 | 0.0871 |
| Dietzia sp. JS16-p6b | 0.010862 | -0.37813 | 0.089611 |
| Dietzia sp. oral taxon 368 | 0.014346 | -0.3643 | 0.098887 |
| Gordonibacter urolithinfaciens | 0.012221 | -0.37233 | 0.091368 |
| Mycobacterium marinum | 0.008272 | -0.39117 | 0.084428 |
| Mycolicibacterium vanbaalenii | 0.010039 | -0.38195 | 0.089069 |
| Plantactinospora sp. KBS50 | 0.003655 | -0.42767 | 0.060361 |
| Rathayibacter toxicus | 0.005213 | -0.41225 | 0.0711 |
| Rhodococcus jostii | 0.009556 | -0.38432 | 0.089069 |
| Streptomyces autolyticus | 0.010891 | -0.378 | 0.089611 |
| Streptomyces coelicolor | 0.013125 | -0.36877 | 0.095382 |
| Streptomyces lincolnensis | 3.88E-06 | -0.64032 | 0.003521 |
| Streptomyces noursei | 0.008272 | -0.39117 | 0.084428 |
| Streptomyces sp. HNM0039 | 0.008507 | -0.38986 | 0.085384 |
| Streptomyces sp. SAT1 | 0.009195 | -0.38617 | 0.088382 |
| Streptomyces venezuelae | 0.000904 | -0.48261 | 0.038102 |
| Aquificae |  |  |  |
| Hydrogenobacter thermophilus | 0.004556 | -0.41818 | 0.066213 |
| Persephonella marina | 0.010345 | -0.3805 | 0.089069 |
| Bacteroidetes |  |  |  |
| Arcticibacterium luteifluviistationis | 0.008919 | 0.387615 | 0.087114 |
| Aureitalea sp. RR4-38 | 0.014495 | 0.363768 | 0.098887 |
| Chryseobacterium indologenes | 0.003225 | 0.432938 | 0.056344 |
| Croceibacter atlanticus | 0.002531 | 0.442951 | 0.052249 |
| Flavobacterium sp. HYN0056 | 0.000307 | 0.519895 | 0.027919 |
| Polaribacter reichenbachii | 0.011189 | 0.37668 | 0.089724 |
| Sediminicola sp. YIK13 | 0.012319 | 0.371937 | 0.091721 |
| Weeksella virosa | 0.002878 | 0.437681 | 0.05565 |
| Winogradskyella sp. PG-2 | 0.001007 | 0.478656 | 0.038913 |
| Chlamydiae |  |  |  |
| Chlamydia abortus | 0.001112 | 0.474967 | 0.040408 |
| Chloroflexi |  |  |  |
| Caldilinea aerophila | 0.00194 | -0.45362 | 0.048422 |
| Roseiflexus castenholzii | 0.002178 | -0.44901 | 0.049464 |
| Roseiflexus sp. RS-1 | 0.005798 | -0.40751 | 0.073656 |
| Cyanobacteria |  |  |  |
| Geminocystis sp. NIES-3709 | 0.008249 | 0.391304 | 0.084428 |
| Gloeobacter kilaueensis | 0.002999 | -0.43597 | 0.05565 |
| Nostoc sp. PCC 7524 | 0.006146 | -0.40487 | 0.075669 |
| Nostocales cyanobacterium HT-58-2 | 0.001802 | 0.456522 | 0.048422 |
| Prochlorococcus marinus | 0.003499 | 0.429513 | 0.05832 |
| Synechococcus sp. JA-3-3Ab | 4.34E-05 | -0.57879 | 0.017794 |
| Synechococcus sp. KORDI-49 | 0.006273 | -0.40395 | 0.07569 |
| Firmicutes |  |  |  |
| Anaerostipes hadrus | 0.007181 | 0.39776 | 0.080043 |
| Bacillus anthracis | 0.009297 | -0.38564 | 0.088893 |
| Bacillus clausii | 0.006291 | 0.403821 | 0.07569 |
| Bacillus cytotoxicus | 0.005781 | 0.407642 | 0.073656 |
| Bacillus kochii | 0.000494 | 0.503953 | 0.031446 |
| Bacillus pumilus | 0.003781 | 0.426219 | 0.060446 |
| Bacillus weihaiensis | 0.000759 | 0.488933 | 0.037892 |
| Blautia hansenii | 0.003735 | 0.426746 | 0.060446 |
| Blautia sp. N6H1-15 | 0.004882 | 0.415152 | 0.068755 |
| Blautia sp. YL58 | 0.009018 | 0.387088 | 0.087614 |
| Brevibacillus laterosporus | 0.007431 | 0.396179 | 0.082054 |
| Cellulosilyticum lentocellum | 0.010177 | 0.381291 | 0.089069 |
| Clostridium argentinense | 0.004709 | 0.416733 | 0.066841 |
| Clostridium clariflavum | 0.007886 | 0.393412 | 0.083039 |
| Clostridium cochlearium | 0.014383 | 0.364163 | 0.098887 |
| Clostridium drakei | 0.003984 | 0.423979 | 0.061346 |
| Clostridium estertheticum | 0.001455 | 0.464822 | 0.047193 |
| Clostridium sp. BNL1100 | 0.013229 | 0.368379 | 0.095754 |
| Clostridium tyrobutyricum | 0.014722 | 0.362978 | 0.099432 |
| Dehalobacterium formicoaceticum | 0.004542 | 0.418314 | 0.066213 |
| Desulfosporosinus meridiei | 0.008602 | -0.38933 | 0.085867 |
| Erysipelothrix rhusiopathiae | 0.006058 | 0.405534 | 0.075669 |
| Eubacterium eligens | 0.003145 | 0.433992 | 0.05565 |
| Eubacterium hallii | 0.003155 | 0.43386 | 0.05565 |
| Eubacterium rectale | 0.002303 | 0.446772 | 0.05041 |
| Ezakiella massiliensis | 0.009795 | 0.383136 | 0.089069 |
| Geobacillus subterraneus | 0.000996 | -0.47905 | 0.038913 |
| Gottschalkia acidurici | 0.006164 | 0.404743 | 0.075669 |
| Lactobacillus casei | 0.007754 | -0.3942 | 0.082378 |
| Lactobacillus murinus | 0.010921 | 0.377866 | 0.089611 |
| Lactobacillus parabuchneri | 0.000853 | 0.484717 | 0.038102 |
| Listeria welshimeri | 0.000187 | 0.535837 | 0.024255 |
| Macrococcus caseolyticus | 0.010012 | 0.382082 | 0.089069 |
| Megamonas hypermegale | 0.002971 | 0.436364 | 0.05565 |
| Oenococcus oeni | 0.010345 | -0.3805 | 0.089069 |
| Paenibacillus beijingensis | 0.01351 | -0.36733 | 0.096252 |
| Paenibacillus sabinae | 0.006218 | -0.40435 | 0.07569 |
| Paenibacillus sp. FSL R5-0345 | 0.008869 | 0.387879 | 0.0871 |
| Planococcus kocurii | 0.007645 | -0.39486 | 0.082182 |
| Selenomonas sputigena | 0.001511 | -0.46337 | 0.047807 |
| Sporosarcina ureae | 0.005418 | 0.41054 | 0.072375 |
| Staphylococcus condimenti | 0.003466 | 0.429908 | 0.058312 |
| Staphylococcus pasteuri | 0.011711 | 0.37444 | 0.090492 |
| Staphylococcus succinus | 0.001148 | 0.473781 | 0.040895 |
| Streptococcus parasanguinis | 0.002087 | 0.450725 | 0.049234 |
| Streptococcus pasteurianus | 0.001914 | 0.45415 | 0.048422 |
| Streptococcus pneumoniae | 0.010261 | 0.380896 | 0.089069 |
| Tepidanaerobacter acetatoxydans | 0.000201 | 0.533597 | 0.024311 |
| Thermincola potens | 0.010572 | -0.37945 | 0.089473 |
| Thermoanaerobacterium thermosaccharolyticum | 0.013369 | 0.367852 | 0.096252 |
| Virgibacillus pantothenticus | 0.004709 | 0.416733 | 0.066841 |
| Bacillus xiamenensis | 0.00573 | 0.408037 | 0.073656 |
| Clostridia Thermoanaerobacterium xylanolyticum | 6.74E-05 | 0.566403 | 0.017794 |
| Fusobacteria |  |  |  |
| Fusobacterium necrophorum | 0.009582 | 0.38419 | 0.089069 |
| Fusobacterium varium | 0.011249 | 0.376416 | 0.089724 |
| Ilyobacter polytropus | 7.02E-05 | 0.565217 | 0.017794 |
| Ignavibacteriae |  |  |  |
| Ignavibacteria Ignavibacterium album | 0.005244 | 0.411989 | 0.0711 |
| Nitrospirae |  |  |  |
| Thermodesulfovibrio yellowstonii | 0.001927 | 0.453887 | 0.048422 |
| Planctomycetes |  |  |  |
| Phycisphaera mikurensis | 0.014533 | -0.36364 | 0.098887 |
| Planctomyces sp. SH-PL62 | 0.00758 | -0.39526 | 0.082182 |
| Proteobacteria |  |  |  |
| Xanthomonas vasicola | 0.012784 | 0.370092 | 0.094277 |
| Acetobacter aceti | 0.010177 | 0.381291 | 0.089069 |
| Acetobacter tropicalis | 0.007161 | -0.39789 | 0.080043 |
| Acidithiobacillus ferrivorans | 0.01131 | -0.37615 | 0.089724 |
| Acinetobacter equi | 0.012818 | 0.36996 | 0.094277 |
| Aeromonas sp. ASNIH2 | 0.000862 | -0.48432 | 0.038102 |
| Allofrancisella guangzhouensis | 0.007953 | 0.393017 | 0.083039 |
| Alteromonas addita | 0.001778 | 0.457049 | 0.048422 |
| Alteromonas sp. MB-3u-76 | 0.01351 | 0.367325 | 0.096252 |
| Aminobacter aminovorans | 0.002597 | -0.4419 | 0.053015 |
| Antarctobacter heliothermus | 0.011555 | -0.3751 | 0.090485 |
| Arcobacter bivalviorum | 0.000142 | 0.544269 | 0.021549 |
| Arcobacter halophilus | 0.014684 | 0.363109 | 0.099432 |
| Bartonella quintana | 0.003096 | 0.434651 | 0.05565 |
| Bordetella bronchialis | 0.01376 | -0.3664 | 0.097269 |
| Bordetella sp. H567 | 0.000914 | -0.48221 | 0.038102 |
| Bordetella sp. HZ20 | 0.001864 | -0.4552 | 0.048422 |
| Bradyrhizobium diazoefficiens | 0.000813 | -0.48643 | 0.037892 |
| Bradyrhizobium sp. BTAi1 | 0.011249 | -0.37642 | 0.089724 |
| Burkholderia sp. JP2-270 | 0.010094 | -0.38169 | 0.089069 |
| Burkholderiales bacterium YL45 | 0.007645 | 0.394862 | 0.082182 |
| Calyptogena okutanii thioautotrophic gill symbiont | 0.001617 | 0.460738 | 0.048041 |
| Campylobacter helveticus | 0.004172 | 0.422003 | 0.062827 |
| Campylobacter sp. RM16704 | 0.000796 | 0.48722 | 0.037892 |
| Candidatus Blochmannia floridanus | 0.006685 | 0.401054 | 0.07785 |
| Candidatus Erwinia sp. ErCipseudotaxifoliae | 0.011009 | -0.37747 | 0.089692 |
| Candidatus Fokinia solitaria | 0.011099 | -0.37708 | 0.089724 |
| Candidatus Kinetoplastibacterium sorsogonicusi | 0.008674 | 0.388933 | 0.086115 |
| Celeribacter ethanolicus | 0.000718 | -0.49091 | 0.037892 |
| Chromobacterium vaccinii | 0.014495 | -0.36377 | 0.098887 |
| Citrobacter koseri | 0.012156 | -0.3726 | 0.091368 |
| Corallococcus coralloides | 0.010572 | -0.37945 | 0.089473 |
| Croceicoccus naphthovorans | 0.000481 | 0.504875 | 0.031446 |
| Cronobacter dublinensis | 0.007645 | -0.39486 | 0.082182 |
| Cystobacter fuscus | 0.00384 | -0.42556 | 0.060514 |
| Desulfobacterium autotrophicum | 0.003689 | -0.42727 | 0.060376 |
| Desulfocapsa sulfexigens | 0.004184 | -0.42187 | 0.062827 |
| Desulfohalobium retbaense | 0.002403 | -0.44506 | 0.05186 |
| Ehrlichia sp. HF | 0.002288 | 0.447036 | 0.05041 |
| Enterobacter sp. FY-07 | 0.00024 | -0.52793 | 0.025635 |
| Enterobacter sp. ODB01 | 0.001223 | -0.47141 | 0.041579 |
| Epibacterium mobile | 0.003864 | -0.4253 | 0.060514 |
| Erwinia billingiae | 0.001236 | -0.47101 | 0.041579 |
| Erythrobacter seohaensis | 0.003793 | 0.426087 | 0.060446 |
| Francisella noatunensis | 0.011868 | 0.373781 | 0.090594 |
| Gammaproteobacteria bacterium DM2 | 0.014533 | 0.363636 | 0.098887 |
| Gammaproteobacteria bacterium ESL0073 | 6.29E-05 | 0.568379 | 0.017794 |
| Gemmobacter sp. HYN0069 | 0.000298 | -0.52095 | 0.027919 |
| Geobacter sp. M18 | 0.001972 | -0.45296 | 0.048422 |
| Geobacter uraniireducens | 0.003423 | -0.43043 | 0.058309 |
| Gluconobacter albidus | 0.000934 | -0.48142 | 0.038102 |
| Halocynthiibacter arcticus | 0.001089 | 0.475758 | 0.040366 |
| Halomonas sp. KO116 | 0.002178 | -0.44901 | 0.049464 |
| Halorhodospira halochloris | 0.003028 | 0.435573 | 0.05565 |
| Helicobacter cetorum | 0.005482 | 0.410013 | 0.072506 |
| Herbaspirillum frisingense | 0.010289 | -0.38076 | 0.089069 |
| Herbaspirillum hiltneri | 0.014346 | -0.3643 | 0.098887 |
| Kangiella koreensis | 0.003067 | -0.43505 | 0.05565 |
| Ketogulonicigenium vulgare | 0.000119 | -0.5498 | 0.019588 |
| Klebsiella oxytoca | 0.013868 | -0.36601 | 0.097277 |
| Klebsiella sp. M5al | 0.010687 | 0.37892 | 0.089473 |
| Kosakonia oryzae | 0.000352 | 0.515415 | 0.028363 |
| Leisingera methylohalidivorans | 0.000748 | -0.48946 | 0.037892 |
| Leminorella richardii | 0.001054 | -0.47694 | 0.039911 |
| Limnobaculum parvum | 0.003434 | 0.430303 | 0.058309 |
| Luteibacter rhizovicinus | 0.000381 | -0.51278 | 0.028839 |
| Mariprofundus aestuarium | 0.008319 | -0.39091 | 0.084428 |
| Massilia armeniaca | 0.01131 | -0.37615 | 0.089724 |
| Mesorhizobium japonicum | 0.00537 | -0.41094 | 0.072267 |
| Methylobacterium phyllosphaerae | 0.001742 | -0.45784 | 0.048422 |
| Methylobacterium sp. C1 | 0.007931 | -0.39315 | 0.083039 |
| Methylocella silvestris | 0.006439 | -0.40277 | 0.076956 |
| Methylococcus capsulatus | 0.005663 | -0.40856 | 0.073487 |
| Methylophilus sp. TWE2 | 0.009984 | 0.382213 | 0.089069 |
| Microvirga ossetica | 0.002426 | 0.444664 | 0.05186 |
| Morganella morganii | 0.01351 | 0.367325 | 0.096252 |
| Moritella yayanosii | 0.004597 | 0.417787 | 0.066285 |
| Myxococcus hansupus | 0.009768 | -0.38327 | 0.089069 |
| Myxococcus xanthus | 0.000284 | -0.52253 | 0.027919 |
| Nitrosomonas sp. Is79A3 | 7.84E-05 | 0.562055 | 0.017794 |
| Obesumbacterium proteus | 0.000359 | 0.514756 | 0.028363 |
| Oblitimonas alkaliphila | 0.011679 | -0.37457 | 0.090492 |
| Octadecabacter antarcticus | 0.00684 | -0.4 | 0.078342 |
| Oleiphilus messinensis | 0.003106 | 0.434519 | 0.05565 |
| Pandoraea pnomenusa | 0.005015 | -0.41397 | 0.070087 |
| Pantoea sp. At-9b | 0.001639 | -0.46021 | 0.048041 |
| Pantoea sp. PSNIH1 | 0.001569 | -0.46192 | 0.048041 |
| Paraburkholderia terrae | 0.004314 | 0.420553 | 0.064239 |
| Paraburkholderia xenovorans | 0.000447 | 0.507378 | 0.031244 |
| Paracoccus aminovorans | 0.000578 | -0.49855 | 0.034995 |
| Paracoccus sp. CBA4604 | 0.006762 | -0.40053 | 0.07825 |
| Phaeobacter inhibens | 0.006646 | -0.40132 | 0.07785 |
| Photobacterium damselae | 0.000173 | 0.53834 | 0.024108 |
| Porphyrobacter neustonensis | 0.003936 | -0.42451 | 0.061115 |
| Proteus mirabilis | 0.000637 | 0.495125 | 0.036179 |
| Providencia rettgeri | 0.010658 | 0.379051 | 0.089473 |
| Pseudoalteromonas phenolica | 0.000502 | 0.503426 | 0.031446 |
| Pseudomonas alcaligenes | 0.001953 | -0.45336 | 0.048422 |
| Pseudomonas azotoformans | 0.009119 | -0.38656 | 0.088118 |
| Pseudomonas cichorii | 0.001864 | -0.4552 | 0.048422 |
| Pseudomonas pseudoalcaligenes | 0.013868 | 0.366008 | 0.097277 |
| Pseudomonas sp. NC02 | 0.005515 | -0.40975 | 0.072506 |
| Pseudomonas stutzeri | 0.002143 | -0.44967 | 0.049464 |
| Pseudomonas syringae | 0.012616 | -0.37075 | 0.093552 |
| Pseudomonas versuta | 0.011493 | 0.375362 | 0.090391 |
| Pseudomonas viridiflava | 1.01E-06 | -0.67167 | 0.001844 |
| Pseudoxanthomonas spadix | 0.011742 | -0.37431 | 0.090492 |
| Psychrobacter sp. P2G3 | 0.001731 | 0.458103 | 0.048422 |
| Psychromonas ingrahamii | 0.011932 | -0.37352 | 0.090697 |
| Psychromonas sp. CNPT3 | 0.010862 | 0.378129 | 0.089611 |
| Rahnella sp. Y9602 | 0.011805 | -0.37404 | 0.090492 |
| Ralstonia pickettii | 0.008021 | 0.392622 | 0.083264 |
| Rhizobacter gummiphilus | 5.84E-05 | -0.57049 | 0.017794 |
| Rhizobium sp. 11515TR | 0.002087 | -0.45072 | 0.049234 |
| Rhodobacter capsulatus | 0.006666 | 0.401186 | 0.07785 |
| Rhodocyclaceae bacterium | 0.006093 | 0.40527 | 0.075669 |
| Rhodomicrobium vannielii | 0.01095 | -0.37773 | 0.089611 |
| Rhodovulum sp. MB263 | 0.006005 | -0.40593 | 0.075669 |
| Rickettsia akari | 0.009426 | -0.38498 | 0.089069 |
| Roseomonas sp. FDAARGOS_362 | 0.009957 | -0.38235 | 0.089069 |
| Serratia ficaria | 0.000802 | -0.48696 | 0.037892 |
| Shewanella bicestrii | 0.002273 | 0.447299 | 0.05041 |
| Shinella sp. HZN7 | 0.003145 | -0.43399 | 0.05565 |
| Sinorhizobium americanum | 0.012092 | -0.37286 | 0.091368 |
| Sinorhizobium fredii | 0.010289 | -0.38076 | 0.089069 |
| Sinorhizobium medicae | 0.004146 | -0.42227 | 0.062827 |
| Sphingobium cloacae | 0.001302 | -0.46904 | 0.043017 |
| Sphingobium indicum | 0.000103 | -0.55415 | 0.01888 |
| Sphingobium sp. TKS | 0.00022 | -0.5307 | 0.024976 |
| Sphingomonas sp. Cra20 | 0.012886 | 0.369697 | 0.094394 |
| Sphingomonas sp. MM-1 | 0.013941 | -0.36574 | 0.09741 |
| Sulfuritalea hydrogenivorans | 0.001612 | -0.46087 | 0.048041 |
| Thiocystis violascens | 0.009876 | -0.38274 | 0.089069 |
| Vibrio harveyi | 0.006685 | -0.40105 | 0.07785 |
| Vibrio rotiferianus | 0.012188 | 0.372464 | 0.091368 |
| Yersinia aleksiciae | 0.000351 | 0.515547 | 0.028363 |
| Yersinia frederiksenii | 0.013581 | 0.367062 | 0.09638 |
| Yersinia ruckeri | 0.00043 | -0.5087 | 0.031244 |
| Spirochaetes |  |  |  |
| Borreliella afzelii | 0.011493 | -0.37536 | 0.090391 |
| Sphaerochaeta globosa | 0.002523 | -0.44308 | 0.052249 |
| Treponema putidum | 0.003423 | 0.430435 | 0.058309 |
| Tenericutes |  |  |  |
| Spiroplasma syrphidicola | 0.004367 | 0.420026 | 0.064496 |
| Tenericutes bacterium MO-XQ | 0.007452 | 0.396047 | 0.082054 |
| Thermotogae |  |  |  |
| Defluviitoga tunisiensis | 0.002726 | 0.439921 | 0.055018 |
| Fervidobacterium nodosum | 0.002066 | 0.45112 | 0.049234 |
| Marinitoga sp. 1137 | 0.009688 | 0.383663 | 0.089069 |
| Petrotoga mobilis | 0.002961 | 0.436495 | 0.05565 |
| Thermosipho melanesiensis | 0.009478 | -0.38472 | 0.089069 |
| Verrucomicrobia |  |  |  |
| Opitutaceae bacterium TAV5 | 0.010658 | -0.37905 | 0.089473 |

Hierarchical All-against-All association testing (HAllA) was used (version 0.8.7) with Spearman correlation as correlation ​metric, medoid as clustering method and q < 0.1 as threshold for significance.

***Supplementary Table 10. Significant correlations between diastolic blood pressure and bacterial species.***

| Bacterial species | p-value | statistic | q-value |
| --- | --- | --- | --- |
| Actinobacteria |  |  |  |
| Arthrobacter sp. U41 | 0.000432 | -0.50856 | 0.085106 |
| Corynebacterium jeikeium | 0.000494 | -0.50395 | 0.085106 |
| Streptomyces lincolnensis | 5.20E-05 | -0.57378 | 0.067098 |
| Cyanobacteria |  |  |  |
| Synechococcus sp. JA-3-3Ab | 0.00044 | -0.50791 | 0.085106 |
| Proteobacteria |  |  |  |
| Rhizobacter gummiphilus | 0.000189 | -0.53544 | 0.081474 |
| Campylobacter sp. RM16704 | 0.000314 | 0.519236 | 0.085106 |
| Gemmobacter sp. HYN0069 | 0.000152 | -0.54216 | 0.078751 |
| Halocynthiibacter arcticus | 0.000126 | 0.54809 | 0.078751 |
| Ketogulonicigenium vulgare | 0.000433 | -0.50843 | 0.085106 |
| Luteibacter rhizovicinus | 0.000449 | -0.50725 | 0.085106 |
| Obesumbacterium proteus | 0.000422 | 0.509354 | 0.085106 |
| Paraburkholderia xenovorans | 0.000569 | 0.499078 | 0.091873 |
| Pseudomonas viridiflava | 8.09E-06 | -0.62253 | 0.020898 |
| Sphingobium indicum | 0.00012 | -0.54941 | 0.078751 |
| Yersinia aleksiciae | 0.000489 | 0.504348 | 0.085106 |
| Yersinia ruckeri | 0.000483 | -0.50474 | 0.085106 |

Hierarchical All-against-All association testing (HAllA) was used (version 0.8.7) with Spearman correlation as correlation ​metric, medoid as clustering method and q < 0.1 as threshold for significance.

***Supplementary table 11. Significant correlations between systolic blood pressure and bacterial species.***

| Actinobacteria | p-value | Statistical | q-value |
| --- | --- | --- | --- |
| Candidatus Rhodoluna planktonica | 0.003246 | 0.432675 | 0.083956 |
| Corynebacterium sp. ATCC 6931 | 0.003466 | -0.42991 | 0.085635 |
| Dietzia sp. oral taxon 368 | 0.003067 | -0.43505 | 0.082224 |
| Mycobacterium ulcerans | 0.000985 | 0.479447 | 0.063073 |
| Pseudarthrobacter chlorophenolicus | 0.002869 | -0.43781 | 0.080105 |
| Renibacterium salmoninarum | 0.004433 | 0.419368 | 0.093723 |
| Sinomonas atrocyanea | 0.00377 | -0.42635 | 0.088936 |
| Streptomyces lincolnensis | 0.002896 | -0.43742 | 0.080105 |
| Streptomyces xiamenensis | 0.001634 | -0.46034 | 0.069792 |
| Bacteroidetes |  |  |  |
| Candidatus Cardinium hertigii | 0.001901 | 0.454414 | 0.070373 |
| Chryseobacterium indologenes | 0.000607 | 0.496838 | 0.063073 |
| Sediminicola sp. YIK13 | 0.004288 | 0.420817 | 0.091804 |
| Caldiserica |  |  |  |
| Caldisericum exile | 0.004262 | 0.42108 | 0.091804 |
| Cyanobacteria |  |  |  |
| Prochlorococcus marinus | 0.002674 | 0.440711 | 0.079433 |
| Deinococcus-Thermus |  |  |  |
| Thermus oshimai | 0.002589 | -0.44203 | 0.077969 |
| Firmicutes |  |  |  |
| Aerococcus urinae | 0.002333 | 0.446245 | 0.075082 |
| Alkaliphilus metalliredigens | 0.000721 | 0.490777 | 0.063073 |
| Anaerostipes hadrus | 0.000739 | 0.489855 | 0.063073 |
| Bacillus clausii | 0.002094 | 0.450593 | 0.070817 |
| Bacillus flexus | 0.00298 | 0.436232 | 0.080897 |
| Bacillus simplex | 0.00128 | 0.469697 | 0.063073 |
| Bacillus weihaiensis | 0.000109 | 0.552306 | 0.039979 |
| Bacillus xiamenensis | 0.001253 | 0.470487 | 0.063073 |
| Blautia hansenii | 0.001298 | 0.46917 | 0.063073 |
| Blautia sp. N6H1-15 | 0.00071 | 0.491304 | 0.063073 |
| Blautia sp. YL58 | 0.001713 | 0.458498 | 0.070373 |
| Clostridium argentinense | 0.001227 | 0.471278 | 0.063073 |
| Clostridium cellulosi | 0.003677 | 0.427404 | 0.088936 |
| Clostridium cochlearium | 0.00091 | 0.482345 | 0.063073 |
| Clostridium drakei | 0.002326 | 0.446377 | 0.075082 |
| Clostridium estertheticum | 0.001435 | 0.465349 | 0.065048 |
| Clostridium pasteurianum | 0.003924 | 0.424638 | 0.088936 |
| Clostridium thermosuccinogenes | 0.002442 | 0.444401 | 0.076707 |
| Dehalobacterium formicoaceticum | 0.00145 | 0.464954 | 0.065048 |
| Eubacterium eligens | 0.001495 | 0.463768 | 0.065748 |
| Eubacterium hallii | 0.00384 | 0.42556 | 0.088936 |
| Eubacterium limosum | 0.002752 | 0.439526 | 0.080105 |
| Eubacterium rectale | 0.001311 | 0.468775 | 0.063073 |
| Geobacillus thermodenitrificans | 0.001348 | 0.467721 | 0.063073 |
| Gottschalkia acidurici | 0.003391 | 0.43083 | 0.084734 |
| Listeria welshimeri | 0.003328 | 0.431621 | 0.084734 |
| Macrococcus canis | 0.001325 | 0.468379 | 0.063073 |
| Mordavella sp. Marseille-P3756 | 0.000207 | 0.532675 | 0.050488 |
| Natranaerobius thermophilus | 0.001156 | 0.473518 | 0.063073 |
| Paenibacillus sp. FSL R5-0345 | 0.001193 | 0.472332 | 0.063073 |
| Paenibacillus sp. lzh-N1 | 0.004752 | -0.41634 | 0.096739 |
| Ruminococcus bicirculans | 0.00176 | 0.457444 | 0.070373 |
| Salimicrobium jeotgali | 0.001914 | 0.45415 | 0.070373 |
| Sporosarcina ureae | 0.000598 | 0.497365 | 0.063073 |
| Staphylococcus succinus | 0.001025 | 0.477997 | 0.063073 |
| Streptococcus agalactiae | 0.001796 | 0.456653 | 0.070373 |
| Streptococcus sp. oral taxon 431 | 0.002841 | 0.438208 | 0.080105 |
| Streptococcus thermophilus | 0.000454 | 0.506851 | 0.063073 |
| Thermoanaerobacter wiegelii | 0.000614 | 0.496443 | 0.063073 |
| Thermoanaerobacterium xylanolyticum | 0.003828 | 0.425692 | 0.088936 |
| Fusobacteria |  |  |  |
| Fusobacterium mortiferum | 0.004681 | 0.416996 | 0.096185 |
| Ilyobacter polytropus | 4.98E-05 | 0.574967 | 0.036504 |
| Sebaldella termitidis | 0.000729 | 0.490382 | 0.063073 |
| Proteobacteria |  |  |  |
| Acetobacter aceti | 0.000699 | 0.491831 | 0.063073 |
| Agrobacterium sp. H13-3 | 0.002012 | -0.45217 | 0.070817 |
| Allofrancisella guangzhouensis | 0.000399 | 0.511199 | 0.062717 |
| Arcobacter bivalviorum | 0.002589 | 0.442029 | 0.077969 |
| Bordetella bronchialis | 0.001808 | -0.45639 | 0.070373 |
| Calyptogena okutanii thioautotrophic gill symbiont | 0.004301 | 0.420685 | 0.091804 |
| Campylobacter hominis | 0.001 | 0.47892 | 0.063073 |
| Candidatus Carsonella ruddii | 0.000841 | 0.485244 | 0.063073 |
| Candidatus Fokinia solitaria | 0.001214 | -0.47167 | 0.063073 |
| Candidatus Nucleicultrix amoebiphila | 0.000146 | 0.543478 | 0.04336 |
| Chromobacterium vaccinii | 0.000657 | -0.49407 | 0.063073 |
| Citrobacter koseri | 0.004009 | -0.42372 | 0.089942 |
| Desulfobacula toluolica | 0.00208 | 0.450856 | 0.070817 |
| Gammaproteobacteria bacterium ESL0073 | 3.93E-05 | 0.581555 | 0.036504 |
| Janthinobacterium sp. 1_2014MBL_MicDiv | 0.004275 | -0.42095 | 0.091804 |
| Klebsiella sp. M5al | 0.004197 | 0.421739 | 0.091804 |
| Lawsonia intracellularis | 0.000319 | 0.518709 | 0.059562 |
| Limnobaculum parvum | 0.004941 | 0.414625 | 0.098755 |
| Magnetospirillum gryphiswaldense | 0.000258 | -0.52556 | 0.056776 |
| Marinobacter hydrocarbonoclasticus | 0.004824 | -0.41568 | 0.097301 |
| Methylophilus sp. TWE2 | 0.001043 | 0.477339 | 0.063073 |
| Myxococcus hansupus | 0.003135 | -0.43412 | 0.08305 |
| Myxococcus xanthus | 0.002356 | -0.44585 | 0.075082 |
| Nitrobacter hamburgensis | 0.002136 | 0.449802 | 0.071139 |
| Obesumbacterium proteus | 0.002915 | 0.437154 | 0.080105 |
| Paraburkholderia phymatum | 0.001999 | -0.45244 | 0.070817 |
| Paracoccus aminovorans | 0.001845 | -0.4556 | 0.070373 |
| Photobacterium damselae | 7.67E-06 | 0.623847 | 0.016863 |
| Pseudoalteromonas phenolica | 0.00112 | 0.474704 | 0.063073 |
| Pseudomonas stutzeri | 0.003246 | -0.43267 | 0.083956 |
| Ralstonia pickettii | 0.003912 | 0.424769 | 0.088936 |
| Rhodospirillum rubrum | 0.002506 | -0.44335 | 0.077615 |
| Rhodovulum sp. MB263 | 0.00192 | -0.45402 | 0.070373 |
| Rickettsia australis | 0.000504 | 0.503294 | 0.063073 |
| Shewanella bicestrii | 0.000393 | 0.511726 | 0.062717 |
| Shewanella sp. WE21 | 0.001152 | -0.47365 | 0.063073 |
| Sphingobium indicum | 0.003381 | -0.43096 | 0.084734 |
| Sphingomonas taxi | 0.000834 | -0.48551 | 0.063073 |
| Wolinella succinogenes | 0.002787 | -0.439 | 0.080105 |
| Kosakonia oryzae | 0.000108 | 0.552569 | 0.039979 |
| Spirochaetes |  |  |  |
| Borreliella afzelii | 0.00091 | -0.48235 | 0.063073 |
| Borreliella burgdorferi | 0.003852 | 0.425428 | 0.088936 |
| Sphaerochaeta coccoides | 0.003888 | 0.425033 | 0.088936 |
| Treponema putidum | 7.29E-05 | 0.564163 | 0.039979 |
| Tenericutes |  |  |  |
| Mycoplasma dispar | 0.000927 | 0.481686 | 0.063073 |
| Thermodesulfobacteria |  |  |  |
| Thermodesulfobacterium commune | 0.004515 | 0.418577 | 0.094532 |
| Thermotogae |  |  |  |
| Fervidobacterium nodosum | 0.001316 | 0.468643 | 0.063073 |
| Petrotoga mobilis | 0.000158 | 0.541107 | 0.04336 |
| Thermotoga profunda | 0.001651 | 0.459947 | 0.069792 |
| Thermotoga sp. Cell2 | 0.002039 | -0.45165 | 0.070817 |
| Thermotogae |  |  |  |
| Opitutaceae bacterium TAV5 | 0.004597 | -0.41779 | 0.095353 |
| Tenericutes |  |  |  |
| Tenericutes bacterium MO-XQ | 0.000325 | 0.51805 | 0.059562 |

Hierarchical All-against-All association testing (HAllA) was used (version 0.8.7) with Spearman correlation as correlation ​metric, medoid as clustering method and q < 0.1 as threshold for significance.
